# Chalcones as a Versatile Antiviral Scaffold: Molecular Targets, ADMET Profiles, and Translational Challenges

**DOI:** 10.3390/v18070806

**Published:** 2026-07-22

**Authors:** Alvaro Luiz Helena, Patrick Rômbola Ozanique, Kevin Henrique Souza Lima, Wellington Negri Tondato, Victor Yukio Ichikawa Baio, Otávio Henrique Locateli Soares, Luis Octávio Regasini

**Affiliations:** Department of Chemistry and Environmental Sciences, Institute of Biosciences, Humanities and Exact Sciences, São Paulo State University (UNESP), São José do Rio Preto 15054-000, SP, Brazil; alvaro.helena@unesp.br (A.L.H.); patrick.ozanique@unesp.br (P.R.O.); kevin.lima@unesp.br (K.H.S.L.); w.tondato@unesp.br (W.N.T.); victor.baio@unesp.br (V.Y.I.B.); otavio.henrique@unesp.br (O.H.L.S.)

**Keywords:** natural products, chalcones, inhibitors, virus, docking, antiviral

## Abstract

Chalcones are naturally occurring open-chain flavonoids widely distributed in plants and recognized for their broad spectrum of pharmacological activities. Their versatile scaffold allows for extensive structural modifications, leading to a diverse range of natural and synthetic derivatives with notable biological potential. In the context of viral infections, chalcones have demonstrated remarkable efficacy against a variety of human pathogens, including dengue virus, HIV, HCV, influenza A, SARS-CoV-2, and other emerging viruses. Beyond human health, several chalcones have shown potent activity against plant viruses such as tobacco mosaic virus (TMV) and cucumber mosaic virus (CMV), and animal viruses including porcine reproductive and respiratory syndrome virus (PRRSV) and mammalian reovirus (MRV), underscoring their broad antiviral spectrum. These compounds act through multiple mechanisms, including the inhibition of viral enzymes (e.g., proteases, polymerases, and integrases), interference with viral entry and replication, and the modulation of host-related pathways. Recent advances in molecular docking, structure–activity relationship (SAR) studies, and synthetic optimization have further highlighted chalcones as a promising scaffold for antiviral drug discovery. Accordingly, this review summarizes and categorizes antiviral chalcones reported over the last two decades, emphasizing and critically discussing their molecular targets, mechanisms of action, and pharmacological potential as lead compounds. It also provides a comparative perspective on their pharmacological relevance by correlating their activities against standard therapeutic agents and reference inhibitors. Furthermore, the most recurrent viral targets were critically discussed regarding their conservation, expected genetic barriers to resistance, and the global SAR trends identified for the corresponding antiviral chalcones. Finally, in silico ADMET profiling of the most promising naturally occurring chalcones was performed to evaluate their drug-likeness and pharmacokinetic properties, offering guidance for future structural optimization and translational development. Collectively, these findings highlight the chalcone scaffold as a versatile platform for the development of novel antiviral agents targeting diverse viral and host pathways.

## 1. Introduction

Natural products and their derivatives play a key role in drug discovery and are a rich source for bioactive compound libraries with a broad spectrum of pharmacological properties [1]. Among these bioactive compounds, chalcones are specialized metabolites widely distributed in plants. These compounds are predominantly biosynthesized by the Asteraceae, Moraceae, Fabaceae, and Aristolochiaceae families, which contribute to defense mechanisms through antiviral, antifungal, antibacterial, anthelmintic, insect antifeedant, insecticidal, and phytotoxic properties. Additionally, chalcones demonstrate important roles in symbiotic interactions, biochemical regulation, pigmentation, and pollination. Besides their ecological functions, chalcones serve as key intermediates in the biosynthesis of flavonoids containing an additional C ring, acting as precursors to various subclasses, including flavones, flavanones, isoflavones, flavonols, anthocyanins, and condensed tannins [2].

**Figure 1:**
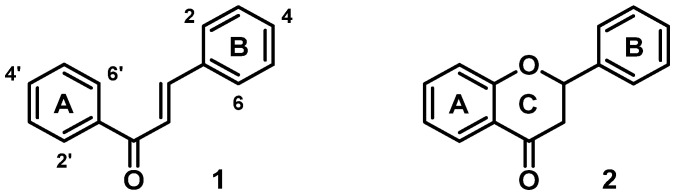
Chemical scaffolds of chalcone (**1**) and flavonoid (**2**).

Chalcones (**1**) are known as open-chain flavonoids (**2**) and are structurally defined by their α,β-unsaturated ketone bridge, which links two aromatic rings A and B (Figure 1) [3,4]. Naturally occurring chalcones commonly bear hydroxyl, methoxy, isoprenyl, and geranyl substituents on rings A and B, with oxidation sites more frequently located on the A ring, particularly at carbons 2′, 4′, and 6′. Altogether, the number and positions of oxygenated substituents are strongly related to their biological activity, as well as their water solubility. Furthermore, the aliphatic chalcone core can act as a Michael acceptor, enabling covalent bonds with thiol groups of cysteine residues, a key property linked to their molecular mechanism of action [5,6,7,8]. Moreover, chalcones can also be synthesized through various chemical reactions, including cross-aldol condensation, cross-coupling, Heck, and Wittig reactions, expanding their accessibility and potential applications [5].

**Figure 2:**
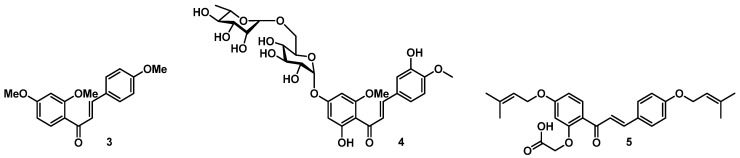
Structures of metochalcone (**3**), hesperidin methyl chalcone (**4**), and sofalcone (**5**).

Beyond their intrinsic ecological and physiological properties against plant pathogens, medicinal plants containing chalcones have been widely used across various traditional medical systems [9]. These compounds exhibit a wide range of pharmacological activities including antioxidant [10], anti-inflammatory [11], antimicrobial [10], anticancer [12,13], antiviral [14], and antileishmanial [15,16] effects. As a reflection of their therapeutic relevance, several naturally occurring chalcones have been approved for market and clinical applications, including metochalcone (**3**), derived from the heartwood of *Pterocarpus marsupium*, known for its choleretic and diuretic effects, and hesperidin methyl chalcone (**4**), found in *Citrus aurantium*, recognized for its vascular protective properties. Additionally, natural-like chalcones, such as sofalcone (**5**), have also contributed significantly to this field, exhibiting anti-ulcer and mucoprotective effects while also demonstrating the ability to inhibit *Helicobacter pylori* growth (Figure 2) [12,13,17].

Viral infections have been responsible for millions of deaths throughout human history, and many of these diseases still lack specific treatments. The recent COVID-19 pandemic, caused by SARS-CoV-2, highlighted the devastating consequences of emerging viruses and the urgent need for effective antiviral therapies. The high mutability of viruses remains a major challenge, often leading to drug resistance and immune evasion, complicating treatment efforts, and contributing to increased mortality rates [14]. In addition to coronaviruses, other viral threats, such as influenza and arboviruses, continue to pose global health risks, reinforcing the importance of discovering novel antiviral agents [18]. In the current review, promising antiviral chalcones were compiled, categorized, and critically discussed to support the development of novel antiviral agents. As a search strategy, relevant articles were retrieved from two primary databases, SciFinder and Web of Science, using the keywords “antiviral” and “chalcone”. The literature search was initially conducted in February 2025 and updated in February 2026 to incorporate newly published studies. The search covered publications from the past 20 years. Studies reporting experimentally determined quantitative antiviral activity (e.g., IC_50_, EC_50_, *K*_i_, *K*_d_, or percentage inhibition) were included in the primary comparative analysis. Docking-only studies were considered separately for mechanistic discussion and are explicitly distinguished throughout the manuscript. Studies involving chalcone-derived scaffolds that did not preserve the canonical chalcone pharmacophore, including cyclized derivatives, carbonyl oxygen replacement, extension or disruption of the α,β-unsaturated ketone bridge, or loss of aromaticity in either ring, were excluded. After applying these eligibility criteria, a total of 56 studies were included. To improve readability, the complete information-rich dataset, including chemical structures and detailed experimental and computational information extracted from all eligible studies, is provided in the Supplementary Materials (Table S1).

## 2. Natural and Synthetic Chalcones Against Arbovirus Infections

Arboviruses are arthropod-borne viruses primarily transmitted by mosquitoes, with *Aedes* and *Haemagogus* genera being the most common vectors. These viruses belong to different families, such as Flaviviridae and Togaviridae, and include numerous pathogens that pose significant public health concerns worldwide [19,20,21]. The spread of arboviral diseases is directly linked to the distribution and adaptability of their vectors. Generally, *Aedes aegypti* is the most common urban vector; however, species with high adaptive capacity, such as *Aedes albopictus*, have contributed to the global expansion of arboviruses. Despite the significant threat they pose to healthcare systems in various countries, many arboviruses remain neglected. This can be attributed to two main factors: first, arboviruses are predominantly endemic to tropical and subtropical regions, which often include low-income, underdeveloped countries with limited economical investment in epidemiological studies, leading to an underestimation of their actual distribution. Second, a large proportion of infections are asymptomatic, making disease surveillance and control even more challenging [22,23]. Currently, no specific antiviral treatments are available for these infections, and therapeutic approaches are limited to supportive care. While some prophylactic agents, such as vaccines, exist for certain arboviruses, many others still lack effective preventive measures [19].

### Dengue, Chikungunya, and Zika Virus

Dengue virus (DENV), Chikungunya virus (CHIKV), and Zika virus (ZIKV) are arboviruses responsible for recurrent outbreaks in tropical and subtropical regions. DENV and ZIKV belong to the Flaviviridae family (genus *Flavivirus*), whereas CHIKV is classified within the Togaviridae family (genus *Alphavirus*). Despite the taxonomic differences, all three are enveloped viruses containing positive-sense single-stranded RNA genomes that encode structural and non-structural proteins essential for viral replication and host interaction [19,21,24]. DENV comprises four antigenically distinct serotypes (DENV-1 to DENV-4) and is the etiological agent of dengue fever (DF), dengue hemorrhagic fever (DHF), and dengue shock syndrome (DSS), with an estimated 50 million infections and approximately 25,000 deaths annually [24,25]. ZIKV gained international attention following large-scale outbreaks exceeding 500,000 confirmed cases and its association with severe neurological complications. Both flaviviruses encode three structural proteins (capsid, pre-membrane, and envelope) and seven non-structural proteins (NS1, NS2A, NS2B, NS3, NS4A, NS4B, and NS5), among which NS3 protease and NS5 polymerase/methyltransferase play central roles in viral replication, whereas structural proteins are involved in host-cell attachment [19,26,27]. In contrast, CHIKV encodes four non-structural proteins (nsP1–nsP4) and five structural proteins (C, E3, E2, 6K, and E1). Clinically, CHIKV infection is characterized by debilitating arthralgia and polyarthralgia, frequently progressing to chronic inflammatory manifestations [21]. Although molecular organization differs between flaviviruses and alphaviruses, their replication strategies share mechanistic similarities, including reliance on viral proteases, RNA-dependent RNA polymerases, and host cellular pathways, making these enzymes attractive targets for small-molecule antiviral development [27].

Few chalcones have been systematically evaluated against dengue virus infections, with most studies focusing exclusively on the DENV-2 serotype, including cardamonin from *Boesenbergia rotunda*, synthetic sulfonamide chalcones, aminochalcones, and sofalcone (Table 1 and Table 2) [28,29,30,31]. While investigations on cardamonin are limited to NS2B/NS3 protease inhibition assays [29], sulfonamide-chalcone derivatives and sofalcone were evaluated against all four dengue serotypes and under both pre- and post-infection conditions, providing a comprehensive antiviral evaluation [30,31]. EC_50_ values ranged from 0.71 to 28.1 µM, highlighting substantial variability in potency depending on structural modifications, cell lineage, and experimental conditions. Moreover, the most potent sulfonamide chalcones were identified as NS5 methyltransferase inhibitors (IC_50_ = 11.58 and 16.85 µM), whereas sofalcone indirectly affected viral replication by modulating the Nrf2-HO-1 signaling pathway, resulting in the inhibition of NS2B/NS3 protease activity (IC_50_ = 10 µM). Sofalcone was the only compound evaluated in vivo, increasing survival rates by approximately 80% at 1 mg/kg in a suckling mouse model, thereby providing translational evidence within this scaffold class [31]. Among the reported studies, molecular docking analyses were scarce, with only one investigation exploring binding interactions at the NS2B/NS3 protease interface. The predicted binding mode suggested stabilization within the catalytic triad region, including a hydrogen bond interaction with Ser135, an essential residue for enzymatic activity [28]. Despite the predominance of NS2B/NS3 as a target in chalcone-based investigations, potent inhibition of the NS5 methyltransferase has also been achieved, suggesting that this enzyme represents a promising alternative target for chalcone scaffold optimization.

On the other hand, chalcone derivatives have been far less explored against CHIKV and ZIKV infections, with the available studies focusing exclusively on naturally occurring scaffolds. For CHIKV, the evidence is restricted to in silico investigations targeting the nsP2 protease, in which angusticornin B, bavachalcone, and licochalcone A (LCA) were identified as promising candidates based on docking energies (*E_dock_* ranging from −115.9 to −95.5 kJ/mol). To assess potential off-target effects, these compounds were also screened against human caspase-3 (HsCASP3). Among them, LCA exhibited the most favorable selectivity profile toward the viral nsP2 protease/HsCASP3 (Table 1) [32], although no experimental validation was performed.

In contrast, the study addressing ZIKV combined in silico predictions with in vitro enzymatic and cellular assays. Three chalcones isolated from *Angelica keiskei* were identified as noncompetitive inhibitors of both the NS2B-NS3 protease complex and the NS5 RNA-dependent RNA polymerase (RdRp) (Table 1). Computational results were consistent with enzymatic assay data, confirming the inhibition of viral protease. Notably, xanthoangelol also inhibited NS5 RdRp with an IC_50_ value of 6.9 µM. However, despite this enzymatic potency, it failed to suppress ZIKV infection in Vero cells at the tested concentrations, which may be associated with its limited aqueous solubility as suggested by the in silico ADMET analysis presented later in this review. Conversely, 4-hydroxyderricin and xanthoangelol-E demonstrated measurable antiviral effects in cell-based assays, with EC_50_ values of 6.6 µM and 22 µM, respectively [33].

Taken together, chalcone scaffolds represent promising starting points for antiviral drug development in the context of arboviral infections. These small molecules have demonstrated the ability to inhibit multiple non-structural viral proteins and offer considerable synthetic versatility, facilitating rational structural modification and systematic structure–activity relationship (SAR) studies. Nevertheless, important gaps remain that hinder translational advancement. Only a limited number of studies have integrated in silico predictions with in vitro and in vivo validation, resulting in fragmented mechanistic and pharmacological characterization. Moreover, no investigation has systematically explored the same conserved molecular target across different arboviruses, despite the high structural similarity among key viral enzymes [27]. Targeting conserved proteins within flaviviruses or alphaviruses may represent a rational strategy toward the development of broad-spectrum anti-arboviral agents, which could significantly strengthen therapeutic readiness and improve clinical management, particularly in regions with high rates of misdiagnosis.

**Table 1:** Antiviral activity of naturally occurring chalcones.

S/N Chalcones	Structure	Virus	Antiviral Activity	Target/Mode of Action	Ref
1—Cardamonin	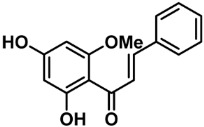	DENV2	*Ki* = 377 ± 77 µM (NS2B-NS3^pro^)	NS2B/NS3 protease (noncompetitive)	[29]
		HCoV-OC43	EC_50_ = 3.62 µM/ SI = >13.81	p38 MAPK signaling pathway	[34]
2—Isoliquiritigenin	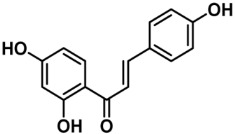	Influenza A	EC_50_ = 24.7 µM/SI = 4.97	PPARγ/Nrf2 pathway; immune regulator	[35]
		Influenza A	IC_50_ = 3.42–9.69 µM (H1N1 and H9N2 NAs)	Neuraminidase (noncompetitive)	[36]
		HCV	IC_50_ = 3.7 µg/mL/SI: 3.0	Not reported	[37]
3—Isobavachalcone	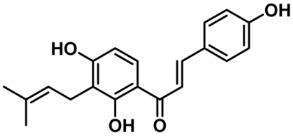	SARS-CoV-2	IC_50_ = 11.9 ± 2.8 µM (3CL^pro^) IC_50_ = 13.0 ± 0.9 µM (PL^pro^)	3CL^pro^ (competitive) PL^pro^ (mixed)	[38]
		PRRSV	IC_50_ = 3.12 µM/SI = 22.02	Viral RNA replication	[39]
4—Xanthohumol	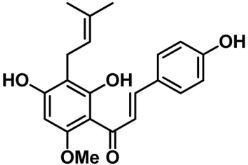	SARS-CoV-2	EC_50_ = 3.3 µM/SI = 3.73 IC_50_ = 162 ± 46 (PL^pro^)	PL^pro^	[40]
5—5-Prenylbutein	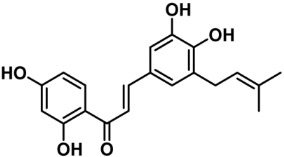	Influenza A	IC_50_ = 25.87–35.50 µM (H1N1 and H9N2 NAs)	Neuraminidase (noncompetitive)	[36]
6—Licoagrochalcone A	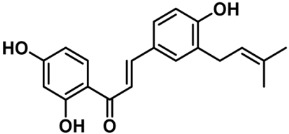	Influenza A	IC_50_ = 51.59–56.92 µM (H1N1 and H9N2 NAs)	Neuraminidase (noncompetitive)	[36]
7—Kanzonol C	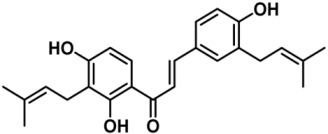	Influenza A	IC_50_ = 52.96–56.92 µM (H1N1 and H9N2 NAs)	Neuraminidase (noncompetitive)	[36]
8—Echinatin	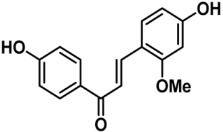	Influenza A	IC_50_ = 2.19–5.80 µM (H1N1 and H9N2 NAs)	Neuraminidase (noncompetitive)	[36]
		SARS-CoV-2	EC_50_ = 7.862 µM/SI = 15.27	Nucleocapsid protein	[41]
9—Licochalcone A	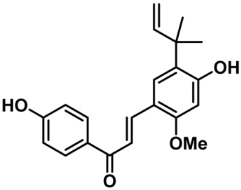	EV-D68	EC_50_ = 3.62 µM/SI = 9.72	Viral IRES-driven translation	[42]
		EV-A71	EC_50_ = 5.73 µM/SI = 5.27	Not reported	[43]
		Influenza A	IC_50_ = 4.20–19.09 µM (H1N1 and H9N2 NAs)	Neuraminidase (noncompetitive)	[36]
		HCV	EC_50_ = 2.5 µg/mL/SI: 8.0	Not reported	[37]
		HSV2	EC_50_ = 1.73 ± 0.04 µM/SI = 13.23	Not reported	[44]
10—Licochalcone B	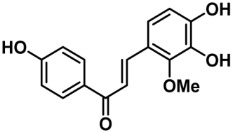	HSV2	EC_50_ = 3.80 ± 0.23 µM/SI = 53.42 EC_50_ = 3.18 ± 0.23 µM/SI = 63.8 (ACV-R)	PI3K-Akt pathway	[44]
		SARS-CoV-2	EC_50_ = 15.53 µM/SI = 6.86	Nucleocapsid Protein inhibitor	[41]
11—Licochalcone D	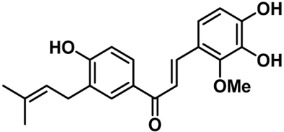	Influenza A	IC_50_ = 28.62–35.21 µM (H1N1 and H9N2 NAs)	Neuraminidase (noncompetitive)	[36]
12—Licochalcone G	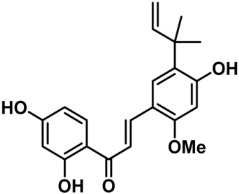	Influenza A	IC_50_ = 37.68–42.11 µM (H1N1 and H9N2 NAs)	Neuraminidase (noncompetitive)	[36]
13—4-Hydroxyderricin	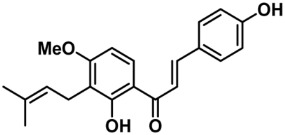	ZIKV	EC_50_ = 6.6 µM/SI: 17 IC_50_ = 47 ± 10 µM (NS2B-NS3^pro^)	NS2B/NS3 protease (noncompetitive)	[33]
		Influenza A	IC_50_ = 42.1 ± 1.8 µM (H1N1 NA)	Neuraminidase (noncompetitive)	[45]
		SARS-CoV-2	IC_50_ = 50.8 ± 3.0 µM (3CL^pro^) IC_50_ = 26.0 ± 1.5 µM (PL^pro^)	3CL^pro^ (competitive) PL^pro^ (noncompetitive)	[38]
14—Xanthoangelol	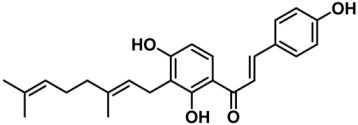	ZIKV	IC_50_ = 50 ± 5 µM (NS2B/NS3^pro^) IC_50_ = 6.9 ± 0.9 µM (NS5^RdRp^)	NS2B/NS3 protease (noncompetitive) and NS5 RdRp	[33]
		SARS-CoV-2	IC_50_ = 5.8 ± 0.6 µM (3CL^pro^) IC_50_ = 11.7 ± 3.2 µM (PL^pro^)	3CL^pro^ (competitive) PL^pro^ (noncompetitive)	[38]
15—Xanthoangelol B	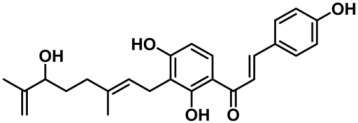	Influenza A	IC_50_ = 22.5 ± 2.2 µM (H1N1 NA)	Neuraminidase (noncompetitive)	[45]
		SARS-CoV-2	IC_50_ = 8.6 ± 2.6 µM (3CL^pro^) IC_50_ = 11.7 ± 0.3 µM (PL^pro^)	3CL^pro^ (competitive) PL^pro^ (noncompetitive)	[38]
16—Xanthoangelol D	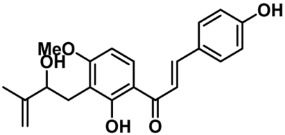	Influenza A	IC_50_ = 12.3 ± 1.1 µM (H1N1 NA)	Neuraminidase (noncompetitive)	[45]
		SARS-CoV-2	IC_50_ = 9.3 ± 1.2 µM (3CL^pro^) IC_50_ = 19.3 ± 1.8 µM (PL^pro^)	3CL^pro^ (competitive) PL^pro^ (noncompetitive)	[38]
17—Xanthoangelol E	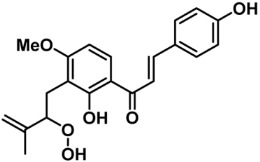	ZIKV	EC_50_ = 22 µM/SI: 5 IC_50_ = 18 ± 5 µM (NS2B/NS3^pro^)	NS2B/NS3 protease (noncompetitive)	[33]
		SARS-CoV-2	IC_50_ = 7.1 ± 0.8 µM (3CL^pro^) IC_50_ = 1.2 ± 0.4 µM (PL^pro^)	3CL^pro^ (competitive) PL^pro^ (noncompetitive)	[38]
18—Xanthoangelol F	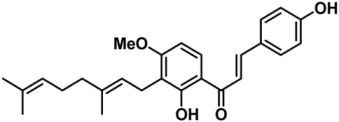	Influenza A	IC_50_ = 85.9 ± 5.0 µM (H1N1 NA)	Neuraminidase (noncompetitive)	[45]
		SARS-CoV-2	IC_50_ = 34.1 ± 4.8 (3CL^pro^) IC_50_ = 5.6 ± 0.5 µM (PL^pro^)	3CL^pro^ (competitive) PL^pro^ (noncompetitive)	[38]
19—Xanthoangelol G	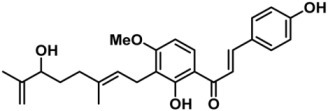	Influenza A	IC_50_ = 24.2 ± 0.7 µM (H1N1 NA)	Neuraminidase (noncompetitive)	[45]
		SARS-CoV-2	IC_50_ = 129.8 ±10.3 (3CL^pro^) IC_50_ = 46.4 ± 7.8 µM (PL^pro^)	3CL^pro^ (competitive) PL^pro^ (noncompetitive)	[38]
20—Xanthokeistal A	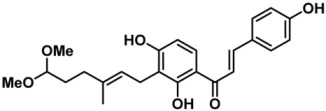	Influenza A	IC_50_ = 30.9 ± 2.1 µM (H1N1 NA)	Neuraminidase (noncompetitive)	[45]
		SARS-CoV-2	IC_50_ = 9.8 ± 2.3 (3CL^pro^) IC_50_ = 21.1 ± 5.6 µM (PL^pro^)	3CL^pro^ (competitive) PL^pro^ (noncompetitive)	[38]
21—6′-O-rhamnosyl-(1‴ → 6″)-glucopyranosyl asebogenin (Thalassodendrone)	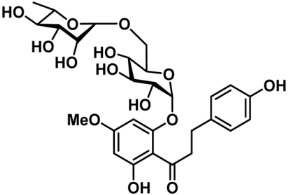	Influenza A	IC_50_ = 2.00 µg/mL/SI: 1.68	Not reported	[46]
22—Asebotin	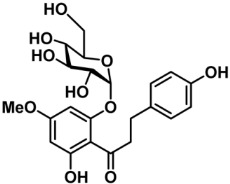	Influenza A	IC_50_ = 1.96 µg/mL/SI: 1.60	Not reported	[46]
23—3-(2-hydroxy-4-methoxyphenyl)-1-(4-hydroxyphenyl)propan-1-one	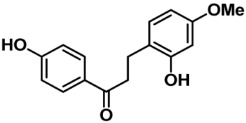	HBV	IC_50_ = 20.56 µg/mL (HBsAg)	HBsAg and HBeAg	[47]
24—(*E*)-3-(2-hydroxy-4-methoxyphenyl)-1-(4-hydroxyphenyl)prop-2-en-1-one	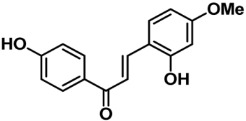	HBV	IC_50_ = 6.36 µg/mL (HBsAg)	HBsAg and HBeAg	[47]
25—Naringenin	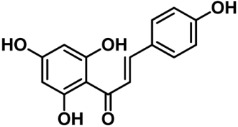	HIV-1	IC50 = 33.0 ± 4.59 µg/mL (HIV-1^pro^)	HIV-1 protease	[48]
26—Kuraridin	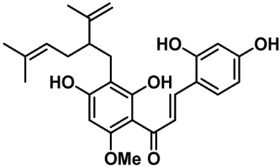	HRV (1–3)	EC_50_ = 14.4–62.0 µM/SI = 4.90–20.98	Sigma-1 protein	[49]

IC_50_ = half-maximal inhibitory concentration; EC_50_ = half-maximal effective concentration; SI = selectivity index (CC_50_/IC_50_); *K*_i_ = inhibition constant.

**Table 2:** Anti-arboviral activity of synthetic chalcones.

S/N Chalcones	Structure	Virus	Antiviral Activity	Target/Mode of Action	Ref
27—SC11	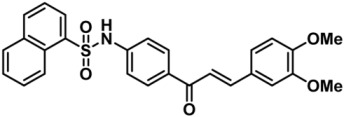	DENV2	EC_50_ = 3.13 ± 0.52 µM/SI = 10.67	Not reported	[30]
28—SC20	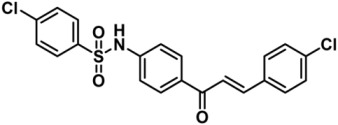	DENV2	EC_50_ = 2.21 ± 0.56 µM/SI = 7.58	Not reported	[30]
29—SC22	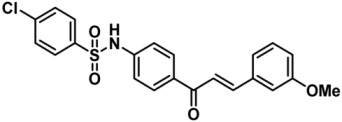	DENV (1–4)	EC_50_ = 0.71–0.94 µM/SI = 15.56–20.60 IC_50_ = 16.85 ± 2.15 µM (NS5^MTase^)	NS5 Methyltransferase	[30]
30—SC24	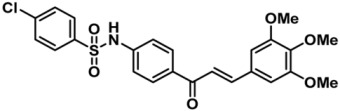	DENV2	EC_50_ = 0.65 ± 0.15 µM/SI = 21.11	Not reported	[30]
31—SC27	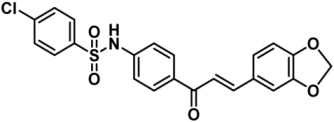	DENV (1–4)	EC_50_ = 3.15–4.46 µM/SI = 6.96–9.85 IC_50_ = 11.58 ± 2.06 µM (NS5^MTase^)	NS5 Methyltransferase	[30]
32—C9	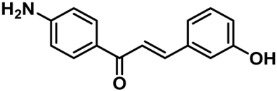	DENV2	IC_50_ = 47.9 µM (NS2B/NS3^pro^)	NS2B/NS3 protease	[28]
33—Sofalcone	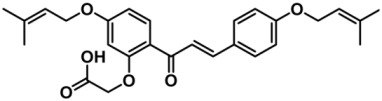	DENV2	EC_50_ = 28.1 ± 0.42 µM IC_50_ = 10 ± 2 µM (NS2B/NS3^pro^)	NS2B/NS3 protease	[31]

IC_50_ = half-maximal inhibitory concentration; EC_50_ = half-maximal effective concentration; SI = selectivity index (CC_50_/IC_50_).

## 3. Chalcones Against Influenza Virus

The influenza virus, a member of the Orthomyxoviridae family, is the causative agent of various flu infections worldwide. It is classified into four types (A, B, C, and D), with influenza A further divided into multiple subtypes based on the genetic variability of its two membrane glycoproteins, hemagglutinin (HA) and neuraminidase (NA). Over time, influenza A viruses have triggered multiple pandemics, including the Spanish flu (H1N1) in 1918, the Asian flu (H2N2) in 1957, the Hong Kong flu (H3N2) in 1968, and the 2009 swine flu (H1N1), resulting in millions of deaths [50]. Influenza A infections can lead to severe clinical complications, including sinusitis, bronchitis, dehydration, pneumonia, encephalitis, and ear infections. The influenza virus is an alphainfluenzavirus with a segmented genome of eight negative-sense single-stranded RNAs that encodes up to 17 proteins, including non-structural proteins (NS), matrix proteins (M), hemagglutinin (HA), neuraminidase (NA), and the RNA-dependent RNA polymerase complex proteins (PB1, PB2, and PA) [50,51,52]. Currently, only a limited number of drugs are available for the treatment of influenza A, with ribavirin being one of the most recently developed. Ribavirin acts as an RNA polymerase inhibitor and exhibits a broad spectrum of antiviral activity, also demonstrating efficacy against HCV and RSV infections. Furthermore, neuraminidase inhibitors, such as zanamivir and its derivatives (oseltamivir, peramivir, and laninamivir octanoate), play a crucial role in influenza treatment, and their design through in silico approaches has revolutionized antiviral drug development [51].

Numerous synthetic and natural chalcone derivatives have been evaluated against multiple influenza A virus subtypes, with the vast majority of studies targeting neuraminidase (NA), particularly from H1N1 strains (Table 1 and Table 3). Structure-based drug design approaches have been widely employed to guide the synthesis of hydroxy-, halo-, and methoxy-substituted chalcones, which were subsequently assessed through enzymatic, cell-based, or a combination of both approaches [53,54,55,56]. Among these efforts, one notable study designed analogs inspired by isoliquiritigenin (ISL), a known NA inhibitor. The most promising ISL-analog exhibited remarkable antiviral potency against H1N1 in MDCK cells (EC_50_ = 1.71 nM) and an exceptionally high selectivity index (SI = 105,597). Enzymatic assays further demonstrated neuraminidase inhibition with IC_50_ values ranging from 3.58 to 12.36 µM, consistent with a noncompetitive mode of action [54]. Interestingly, the marked discrepancy between enzymatic and cellular potency suggests that additional antiviral mechanisms beyond direct NA inhibition may contribute to the observed activity, underscoring the need to explore alternative viral or host-related targets.

An additional noteworthy study focused on oseltamivir-resistant H1N1 (pdm09) strain inhibition. In this context, a chalcone containing a 2′,6′-dimethoxyphenyl moiety exhibited an EC_50_ of 1.34 µM and moderate cytotoxicity in chicken embryo fibroblast (CEF) cells (CC_50_ = 41.46 µM). Subsequent structural optimizations yielded a derivative with comparable antiviral potency and an improved cytotoxicity profile (CC_50_ > 100 µM), underscoring the potential of chalcones as a versatile scaffold for anti-influenza drug development [56].

**Figure 3:**
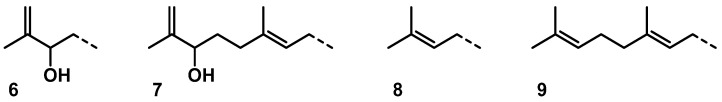
Structures of substituents 2-hydroxy-3-methyl-3-butenyl alkyl (**6**), 6-hydroxy-3,7-dimethyl-octa-2,7-dienyl (**7**), dimethylallyl (**8**), and geranyl (**9**).

Naturally occurring chalcones isolated from *Glycyrrhiza inflata*, *Angelica keiskei*, and *Thalassodendron ciliatum (Forsk.) den Hartog* were predominantly investigated as neuraminidase (NA) inhibitors against influenza A strains (Table 1) [35,36,45,46]. Across these independent studies, IC_50_ values ranged from 5.7 to 85.9 µM against H1N1 and H9N2 NA, with licochalcone A, echinatin, isoliquiritigenin (ISL), and xanthoangelol D among the most potent representatives. Notably, kinetic analyses consistently demonstrated a noncompetitive mode of inhibition, suggesting interaction with allosteric sites (distinct from the NA catalytic pocket targeted by oseltamivir) [36,45]. Within the *G. inflata* series, non-prenylated chalcones exhibited superior inhibitory activity, and echinatin showed pronounced synergism with oseltamivir, enhancing inhibition of the H274Y-resistant NA mutant by 52.6-fold [36]. Although alkylation is often associated with reduced activity, the *A. keiskei* series enabled a more nuanced evaluation of these substituent effects. In this case, the potency followed the order: 2-hydroxy-3-methyl-3-butenyl alkyl (6) > 6-hydroxy-3,7-dimethyl-octa-2,7-dienyl (7) > dimethylallyl (8) > geranyl (9) (Figure 3), underscoring the influence of side-chain structure on allosteric NA inhibition [45]. Beyond direct NA inhibition, ISL showed additional antiviral and immunomodulatory effects, with an EC_50_ of 24.7 µM in H1N1-infected Calu-3 cells and in vivo efficacy using a phosphate prodrug (10 mg/kg), resulting in reduced weight loss and decreased pulmonary CD8^+^ T-cell infiltration. Given the central role of excessive inflammation in influenza pathogenesis, the immunomodulatory properties of ISL were investigated, revealing activation of the PPARγ pathway. The compound was proposed to function as a dual PPARγ and Nrf2 agonist, thereby combining antiviral, anti-inflammatory, and antioxidant effects. These pleiotropic activities contributed to reduced viral load, decreased inflammatory cytokine expression, and diminished immune cell recruitment, highlighting PPARγ modulation as a complementary therapeutic strategy in influenza management [35].

Collectively, although chalcone derivatives represent an important class of neuraminidase (NA) inhibitors, current research remains largely target-restricted. The predominance of NA-centered investigations overlooks other essential viral enzymes, such as the polymerase complex (PA/PB1/PB2) and the M2 ion channel [57], as well as host-dependent pathways that contribute to viral replication and pathogenesis. Furthermore, the immunomodulatory properties observed for certain chalcones suggest that antiviral efficacy may extend beyond direct enzymatic inhibition, highlighting the need for integrated mechanistic studies that simultaneously evaluate antiviral and host-response modulation. Future efforts should therefore prioritize target diversification, systematic structure–activity relationship expansion across multiple viral proteins, and comprehensive in vitro–in vivo correlation studies to fully elucidate the therapeutic potential of this scaffold.

## 4. Chalcones Against TMV and CMV Virus

Tobacco mosaic virus (TMV) and cucumber mosaic virus (CMV) are single-stranded RNA viruses that represent two of the most widespread and economically significant plant pathogens. TMV, the type species of the *Tobamovirus* genus, primarily infects solanaceous crops such as tobacco, tomato, and potato, causing characteristic mosaic patterns, leaf deformation, and growth inhibition. In contrast, CMV belongs to the *Cucumovirus* genus and has one of the broadest host ranges among plant viruses, infecting over 1300 plant species, including cucurbits, legumes, and ornamentals. Both viruses contribute to severe agricultural losses worldwide by reducing crop yield and quality. Due to their well-characterized genomes and transmission mechanisms, TMV and CMV are frequently used as model systems for studying plant–virus interactions and the evaluation of novel antiviral agents [58,59,60,61].

In this context, chalcone-based derivatives have been extensively investigated through hybridization with purine, oxadiazole/thiadiazole, triazole, pyridine, malonate, aminophosphonate, phenoxypyridine, and other heterocyclic fragments [59,60,61,62,63,64,65,66,67,68,69,70,71,72] (Table 4). Despite the chemical diversity of these hybrid chalcones, a notable mechanistic convergence is observed: most active compounds target the TMV coat protein (TMV-CP), a highly conserved structural protein essential for virion assembly and stability [73,74]. Several derivatives displayed promising activity profiles, including a purine-hybrid chalcone (EC_50_ = 51.7 µg/mL against TMV; 89% inactivation at 500 µg/mL) [61], oxadiazole/thiadiazole hybrids (EC_50_ = 30.57–33.97 µg/mL) [59], and a triazole-fused analog (curative EC_50_ = 77.64 µg/mL; 88% inactivation at 500 µg/mL) [63]. Binding studies consistently reported micromolar affinity (*Kd* ≈ 5–6 µM) toward TMV-CP, supported by molecular docking and fluorescence titration assays, and in some cases corroborated by electron microscopy analyses indicating viral particle disruption [59,61,68]. In contrast, only a few naturally occurring chalcones have been explored, including chalcones from *Rosa rugosa* and two chalconoids from *Phyllanthus emblica* [75,76]. Among them, only one chalcone from *R. rugosa* stood out, displaying an IC_50_ of 52.1 μM against TMV (comparable to the positive control, ningnanmycin), although no mechanistic validation was performed [75] (Table 1).

Structurally, the recurrent interaction with TMV-CP suggests that chalcones may interfere with protein–protein interfaces critical for helical assembly, thereby potentially destabilizing virion architecture [73]. However, this strong focus on a single structural protein also highlights a central limitation: alternative viral targets involved in replication or RNA synthesis remain largely unexplored. Moreover, although certain derivatives enhanced host defense markers, such as increased peroxidase and catalase activities or reduced malondialdehyde accumulation [64,68,71], the molecular pathways underlying these responses are not completely characterized. Another gap lies in the fragmented SAR interpretation across independent chemical series, with limited cross-comparison of substituent effects or quantitative structure-binding correlations. Future research should therefore integrate high-resolution structural studies of chalcone/TMV-CP complexes, expand target exploration to viral replication-associated proteins, and establish systematic SAR frameworks that correlate physicochemical properties with both binding affinity and in vivo antiviral efficacy. Such advances would enable the rational development of more potent and mechanistically defined anti-TMV/CMV chalcone derivatives.

## 5. Chalcones Against Hepatitis B and C Virus

Hepatitis B virus (HBV), a member of the Hepadnaviridae family, is a small DNA virus that attacks the liver, inducing hepatitis B infection and is commonly transmitted during birth from mother to child. Whereas HBV typically leads to an acute, short-term infection, it can also induce a chronic phase, which is associated with cirrhosis and liver cancer [77]. It was estimated that 254 million people were living with chronic hepatitis B infection by 2022. The same year, hepatitis B resulted in an estimated 1.1 million deaths, mostly related to the chronic form of infection. Vaccination remains the best preventive strategy against hepatitis B; although there is no specific treatment for the acute phase, chronic hepatitis B is managed with palliative approaches aimed at slowing the advance of cirrhosis and improving long-term survival with the use of oral medicines such as tenofovir or entecavir [78].

Hepatitis C virus (HCV) is a member of the Flaviviridae family and is the causative agent of hepatitis C. This virus causes both acute and chronic hepatitis, which ranges from mild illness to chronic and self-limiting conditions, such as liver cancer. The virus is transmitted through exposure to contaminated blood, including contaminated sharps (e.g., needles), or sexual exposure without barrier protection [79]. It is estimated that 50 million people have the chronic form of hepatitis C infection worldwide and it was estimated that 242,000 people died from hepatitis C in 2022, mostly from chronic infection. Whereas there is no effective vaccine available, the treatment with direct-acting antiviral medicines (DAA) shows a cure rate of 95% of people with the infection, although access to diagnosis and treatment remains limited [80].

Within this framework, the available evidence is largely restricted to naturally occurring chalcones. Collectively, these reports indicate that this scaffold displays measurable activity against both the Hepatitis B virus and Hepatitis C virus; however, the findings diverge considerably in terms of mechanistic insight, potency profiles, and therapeutic positioning (Table 1). In HBV models, the chalcones from *Dracaena cinnabari* demonstrated relatively favorable therapeutic indices (TI up to 38) and moderate suppression of viral DNA replication, even outperforming lamivudine in short-term assays, suggesting a direct antiviral effect at the level of antigen expression and replication [47]. In contrast, studies involving xanthohumol primarily focused on hepatoprotection and the modulation of oxidative stress and apoptosis in HCV-infected animals, without clearly establishing the inhibition of viral replication itself [81]. Meanwhile, ISL and LCA showed direct post-entry anti-HCV activity in vitro, with IC_50_ values of 3.7 and 2.5 μg/mL, respectively, although their selectivity indices remained modest (SI = 3–8) [37], indicating a limited therapeutic window. The most relevant molecular targets identified were viral antigen production (HBsAg/HBeAg) and post-entry replication stages in HCV; however, none of the studies conclusively defined specific viral enzymes (e.g., HBV polymerase, HCV NS3/NS5B) or host factors as primary molecular targets.

The principal gaps therefore include the lack of target-based mechanistic validation, limited structure–activity relationship (SAR) integration across independent studies and scarce in vivo antiviral efficacy data beyond hepatoprotective endpoints. Overall, while these works establish natural chalcones as a promising antiviral scaffold with multitarget potential, systematic mechanistic elucidation and rational optimization remain essential to advance them from preliminary bioactive compounds to clinically relevant antiviral leads.

## 6. Chalcones Against HIV

Human immunodeficiency virus (HIV), a member of the Retroviridae family, is the causative agent of acquired immunodeficiency syndrome (AIDS) and classified in two main genotypes, HIV-1 and HIV-2, with HIV-1 being responsible for the majority of the global HIV/AIDS pandemic [82]. According to projections from the 2024 UNAIDS report, if HIV programs continue at the same pace as in 2022, an estimated 46 million people will be living with HIV worldwide by 2050 [83]. The HIV genome comprises three major genes essential for viral replication: *gag*, which encodes structural proteins; *pol*, which encodes viral enzymes reverse transcriptase (RT), integrase (IN), and protease (PR); and *env*, which encodes the envelope glycoproteins required for viral entry [84]. The current treatment for HIV/AIDS relies on antiretroviral therapy (ART). According to the 2024 UNAIDS report, approximately 89% of individuals who were aware of their HIV status were receiving ART, and among these, 93% had achieved viral suppression in 2023 [83]. While ART has been highly effective in slowing disease progression and reducing viral transmission, its long-term use is frequently associated with adverse side effects. Moreover, ART long-term users experience higher rates of chronic comorbidities such as cardiovascular disease, hypertension, diabetes, and depression, as well as increased risks, due to HIV itself, of cancer and immune dysfunction compared to the general population [83,85].

Collectively, the reported studies demonstrate that chalcone derivatives have been explored against multiple validated HIV-1 and HIV-2 molecular targets, predominantly reverse transcriptase (RT), integrase (IN), and protease (PR), reflecting a rational alignment with established antiretroviral strategies (Table 1 and Table 5) [48,82,86,87,88]. However, the degree of mechanistic validation and translational robustness varies considerably across compound classes.

Among these targets, RT inhibition appears to be the most consistent strategy. Quinoline-containing chalcones achieved potent antiviral activity in PBM cells (EC_50_ = 1.43–8.12 μM) and EC_90_ across three cellular models (PBM, CEM, and Vero cells), accompanied by strong enzymatic inhibition of RT for the most active compounds (IC_50_ = 0.10–0.11 μg/mL). These findings suggest effective engagement of the hydrophobic pocket of RT, further supported by molecular docking analyses that corroborated the observed enzymatic potency [86]. Additional quinolinyl derivatives maintained EC_50_ values below 5 μM, reinforcing RT as a responsive target for chalcone-based scaffolds [88]. Despite these encouraging potency profiles, kinetic evaluations and resistance mapping against clinically relevant reverse-transcriptase inhibitor (RTI) resistant strains were not performed, leaving their true therapeutic positioning uncertain.

Integrase-targeted chalcones demonstrated moderate antiviral activity (EC_50_ ≈ 7–9 μM) and provided mechanistic resolution at the catalytic level, with selective inhibition of strand transfer and dual inhibition of 3′-processing and strand transfer steps. This catalytic-step selectivity is mechanistically relevant given the clinical success of strand-transfer inhibitors [87]; however, antiviral potency remained inferior to that observed for RT-directed derivatives. In contrast, protease inhibition was comparatively weak. Naringenin chalcone displayed only modest enzymatic inhibition (IC_50_ = 33 μg/mL), substantially lower than reference inhibitors, and no compelling cellular antiviral data were presented [48]. Similarly, chalcone-based imidazo[1,2-a]pyridine derivatives exhibited favorable docking scores against RT (HIV-1) or PR (HIV-2) but failed to translate into meaningful in vitro antiviral efficacy (SI ≤ 1), highlighting the recurrent disconnect between computational predictions and biological validation [82]. Additionally, other synthetic chalcones demonstrated high percentages of viral inhibition at fixed concentrations (10 μM) and acceptable cytotoxicity profiles; however, the absence of detailed EC_50_ values and precise molecular target identification limits mechanistic interpretation and cross-comparison [85].

Overall, chalcone scaffolds exhibit micromolar to low-micromolar antiviral activity, particularly against RT and IN, supporting their classification as viable lead-like structures. Nevertheless, advancement from exploratory antivirals to clinically relevant candidates will require systematic evaluation against resistant strains, comprehensive target-specific mechanistic studies, optimized SAR development, and in vivo pharmacological validation within combination therapy frameworks.

## 7. Chalcones Against Coronavirus

*Betacoronavirus* (β-CoVs), a genus within the Coronaviridae family, comprises pathogens responsible for a wide range of respiratory syndromes [89]. Among them, severe acute respiratory syndrome coronavirus 2 (SARS-CoV-2) has caused one of the most devastating health crises in human history, the COVID-19 pandemic, which has resulted in over 676 million infections and nearly seven million deaths [40,89]. The genus also includes other clinically relevant viruses, such as SARS-CoV-1 and Human Coronavirus OC43 (HCoV-OC43), both of which are frequently used in research and drug development as surrogate models for SARS-CoV-2 due to their high genomic similarities [90]. SARS-CoV-2 is an enveloped, positive-sense, single-stranded RNA virus that encodes four major structural proteins: spike (S), membrane (M), envelope (E), and nucleocapsid (N). While the M, E, and N proteins primarily contribute to the virus’s architecture and assembly, the spike glycoprotein plays a pivotal role in host cell recognition and entry through interaction with the human angiotensin-converting enzyme 2 (ACE2) receptor [89,91]. Consequently, both the S protein and ACE2 have emerged as central molecular targets for strategies designed to block viral entry and infection [92]. Beyond structural components, the SARS-CoV-2 genome encodes two large polyproteins (pp1a and pp1ab), which are translated upon viral entry and subsequently cleaved by two viral cysteine proteases: the 3-chymotrypsin-like protease (3CL^pro^, also known as the main protease, M^pro^) and the papain-like protease (PL^pro^) [92,93]. This proteolytic processing yields 16 nonstructural proteins that form the replication-transcription complex, which is essential for viral RNA synthesis and replication. Both 3CL^pro^ and PL^pro^ have been widely recognized as promising antiviral targets due to their crucial role in the viral life cycle [38,94]. The main therapeutic strategies for COVID-19 currently include nirmatrelvir (administered with ritonavir as *Paxlovid*), a reversible inhibitor of the SARS-CoV-2 main protease (3CL^pro^) with an EC_50_ of 74.5 nM; remdesivir (*Veklury*), an inhibitor of the viral RNA-dependent RNA polymerase (RdRp) with an EC_50_ of 37 ± 0.4 nM; and monoclonal antibodies (mAbs), such as bebtelovimab, regdanvimab, and sotrovimab, which block the interaction between the spike protein and the ACE2 receptor [40,95]. While viral replication inhibitors exhibit a relatively low propensity for resistance development, mAbs have lost most of their neutralizing activity against emerging SARS-CoV-2 variants, particularly the Omicron sublineages. This loss of efficacy has markedly limited therapeutic options for patients within the first 10 days of symptom onset, underscoring the urgent need for continued research and development of new anti-SARS-CoV-2 agents [95].

Chalcone derivatives have been investigated against SARS-related coronaviruses through multiple mechanistic strategies, including the direct inhibition of viral proteases [38,40,89,91,92,93,96], interference with host entry factors [92,97], and modulation of host antiviral pathways (Table 1 and Table 6) [34,41,91]. Among these approaches, viral proteases, particularly the main protease (3CL^pro^/M^pro^) and papain-like protease (PL^pro^), have emerged as the most extensively explored targets due to their essential role in viral polyprotein processing and replication.

Natural chalcones isolated from *A. keiskei* represent one of the earliest systematic investigations of this scaffold against coronavirus proteases. These compounds inhibited SARS-CoV 3CL^pro^ with IC_50_ values ranging from 11.4 to 129.8 μM in both cell-free and cell-based cleavage assays, acting through competitive inhibition. On the other hand, inhibition of PL^pro^ occurred through a noncompetitive mechanism with improved potency (IC_50_ = 1.2–46.4 μM), suggesting that chalcones with these structural features may preferentially interact with allosteric or other noncatalytic regions of the enzyme. Within this series, xanthoangelol E (distinguished by the presence of a perhydroxyl substituent) emerged as the most potent dual inhibitor of both viral proteases. Additional enzymatic screening revealed a marked selectivity toward cysteine proteases, suggesting that this scaffold may be particularly suitable for targeting viral proteolytic machinery while minimizing off-target effects on serine proteases [38]. In contrast, another alkylated chalcone (xanthohumol) exhibited moderate PL^pro^ inhibition in vitro but demonstrated greater antiviral potency on phenotypic assays (EC_50_ = 3.3 μM) [40], consistent with previous reports describing this compound as a potential broad-spectrum inhibitor of the highly conserved M^pro^ across multiple coronaviruses [98].

More recent studies have expanded protease-targeted strategies through synthetic derivatives. Catechol-containing chalcones inspired by the natural product danshensu displayed strong inhibition of SARS-CoV-2 3CL^pro^, achieving nanomolar enzymatic potency (IC_50_ = 83.2–261.3 nM) alongside measurable antiviral activity in replicon assays (EC_50_ = 11.7–19.9 μM). These results highlight the catechol moiety as a promising pharmacophore for protease binding and enzymatic inhibition [93]. Computational analyses further support this structural motif, as exemplified by the predicted interactions of broussochalcone A with the 3CL^pro^ of both SARS-CoV-1 and SARS-CoV-2 [89]. Collectively, these findings reinforce the suitability of chalcone scaffolds as an inhibitor of coronavirus cysteine proteases and provide a basis for future structural optimization.

Complementary virtual screening studies have also proposed several chalcone-based derivatives, including hybrid and acetamide-functionalized analogs [91,92,96]. Beyond targeting viral proteases, these compounds were predicted to interact with additional viral or host-related targets such as host-based antiviral proteins (HBATs), viral methyltransferase, and the spike-ACE2 interface [91,92]. Such multitarget profiles suggest that structurally diversified chalcones may offer opportunities to disrupt multiple stages of the viral life cycle simultaneously. However, the absence of enzymatic assays or phenotypic antiviral validation for most of these candidates highlights a persistent limitation of docking-centered studies.

In addition to protease inhibition, several chalcones appear to exert antiviral activity in vitro through host-related mechanisms. Cardamonin, for instance, inhibited the replication of the human coronavirus HCoV-OC43 (IC_50_ = 3.62 μM, SI > 13.8) by activating the p38 MAPK signaling pathway, indicating that host-directed immunomodulatory mechanisms may contribute to its antiviral activity [34]. Similarly, echinatin and licochalcone B, identified through the screening of traditional Chinese medicine compounds, demonstrated antiviral activity against SARS-CoV-2 in Vero E6 cells (EC_50_ = 7.86 and 15.53 μM, respectively) and primarily affected the post-entry stage of infection, markedly suppressing viral nucleocapsid protein expression [41]. In addition, 4-hydroxychalcone showed potent antiviral activity against HCoV-OC43 in RD cells (IC_50_ = 1.83 μM), most likely suppressing viral replication through inhibition of the EGFR/AKT/ERK1/2 signaling pathway. Notably, in vivo evaluation in a suckling mouse model demonstrated a 75% increase in survival rate at 20 mg/kg, accompanied by reduced levels of pro-inflammatory cytokines and chemokines [99]. Collectively, these findings indicate that chalcones may modulate intracellular replication processes in addition to directly targeting viral enzymes. Other studies have explored interference with viral entry mechanisms; for example, docking analyses identified the catechol-chalcone butein as a potential binder of the ACE2 receptor with favorable predicted pharmacokinetic properties [97], although experimental confirmation of its functional relevance remains necessary.

Taken together, these studies highlight the remarkable structural versatility of chalcone scaffolds, which enables interaction with multiple components of the coronavirus replication cycle, including viral proteases, host receptors, and intracellular signaling pathways. Among the proposed mechanisms, inhibition of viral cysteine proteases, particularly 3CL^pro^, currently provides the most robust foundation for antiviral development, especially when supported by both enzymatic and cell-based validation. Nevertheless, several critical gaps remain. A substantial portion of reported candidates arises from computational predictions that lack biochemical or phenotypic confirmation, while heterogeneous experimental models and incomplete pharmacological reporting hinder direct comparison across studies. Furthermore, systematic structure–activity relationship analyses, resistance profiling against emerging variants, and in vivo antiviral evaluation are still scarce. Addressing these limitations through integrated medicinal chemistry, standardized antiviral assays, and translational validation will be essential to advance chalcone-based compounds from exploratory antiviral scaffolds toward viable therapeutic leads against SARS-related coronaviruses.

**Table 6:** Anti-CoVs activity of synthetic chalcones.

S/N Chalcones	Structure	Virus	Antiviral Activity	Target/Mode of Action	Ref
87—A4	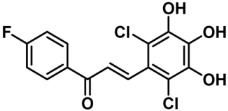	SARS-CoV-2	IC_50_ = 83.2 nM (3CL^pro^) EC_50_ = 19.9 µM (Replicon)	3CL protease (mixed)	[93]
88—A7	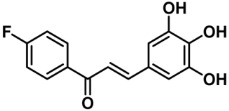	SARS-CoV-2	IC_50_ = 261.3 nM (3CL^pro^) EC_50_ = 11.7 µM (Replicon)	3CL protease (competitive)	[93]
89—4-hydroxychalcone	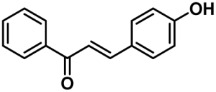	HCoV-OC43	EC_50_ = 1.83 ± 0.17 µM/SI = 13.46	EGFR/AKT/ERK1/2 signaling pathway	[99]

IC_50_ = half-maximal inhibitory concentration; EC_50_ = half-maximal effective concentration; SI = selectivity index (CC_50_/IC_50_); *K*_i_ = inhibition constant.

## 8. Miscellaneous

Beyond the viral systems discussed in previous sections, chalcone derivatives have also demonstrated antiviral activity against several additional RNA and DNA viruses (Table 1). Although these studies are relatively limited and often focused on individual compounds, they further highlight the structural versatility of the chalcone scaffold and its ability to interfere with diverse stages of viral replication.

Several chalcones have been reported to inhibit phylogenetically diverse RNA viruses, including enteroviruses and reoviruses. Among them, licochalcone A (LCA) showed notable activity against enteroviruses associated with neurological and respiratory diseases. In cell-based assays, LCA inhibited enterovirus A71 (EV-A71) with EC_50_ values of 9.30 μM in RD cells and 5.73 μM in Vero cells while also suppressing additional enterovirus strains such as EV-A71 C4, EV-A71 H, and coxsackievirus A16. Time-of-addition experiments indicated that LCA primarily acts during the early phase of infection, and in vivo studies using neonatal mice demonstrated reduced viral loads in the brain, intestine, and hind-limb muscle following treatment [43]. Similarly, LCA inhibited enterovirus D68 (EV-D68), displaying an EC_50_ of 3.62 μM and a selectivity index of 9.72. Mechanistic investigations suggested that this activity is associated with the suppression of viral protein synthesis through inhibition of the internal ribosome entry site (IRES), resulting in marked reductions in IRES activity for EV-D68, EV-A71, and coxsackievirus B3 [42].

Other chalcones have also demonstrated antiviral activity against distinct RNA viruses. Isobavachalcone (IBC), for example, exhibited potent inhibition of porcine reproductive and respiratory syndrome virus (PRRSV), with an IC_50_ of 3.12 μM and a selectivity index of 22.02 in MARC-145 cells. Although initial assays suggested early-stage inhibition, further mechanistic studies indicated that IBC does not affect viral attachment or entry but instead suppresses viral RNA replication, as evidenced by reduced intracellular double-stranded RNA levels [39]. In addition, kuraridin, a chalcone isolated from *Sophora flavescens*, displayed inhibitory effects against mammalian reoviruses infecting both human and porcine hosts. Antiviral assays revealed EC_50_ values ranging from 14.0 to 176.9 μM depending on the viral strain, with mechanistic analyses indicating that kuraridin interferes with viral entry through the inhibition of sigma-1 mediated adsorption and also reduces viral RNA levels during infection [49].

Chalcones have also demonstrated activity against DNA viruses, particularly herpes simplex virus (HSV), a clinically important pathogen capable of establishing latent infections in nervous tissue. Among the compounds evaluated, LCA and licochalcone B (LCB) showed significant inhibitory effects against HSV-2 in Vero cells, with IC_50_ values of 1.73 μM and 3.80 μM, respectively. Notably, LCB displayed a high selectivity index (53.42) and retained activity against acyclovir-resistant HSV-2 strains. Mechanistic studies suggested that this compound suppresses viral replication by reducing glycoprotein D (gD) expression and modulating host signaling pathways, particularly through the downregulation of Akt phosphorylation. Pre-incubation assays further indicated that LCB may interact directly with viral particles. Importantly, in vivo evaluation using a murine vaginal infection model demonstrated that treatment with LCB significantly improved body-weight recovery, showing effects comparable to those observed with acyclovir treatment [44].

Although these studies involve a limited number of viral models, they collectively reinforce the broad antiviral potential of chalcone derivatives across phylogenetically unrelated viruses. Importantly, the reported mechanisms include the inhibition of viral entry, suppression of RNA replication, and interference with viral protein synthesis or host signaling pathways. Nevertheless, most investigations remain restricted to individual compounds and early-stage experimental models, highlighting the need for more systematic structure–activity relationship studies and comprehensive mechanistic validation.

## 9. Comparison of the Antiviral Activity of Chalcones with That of Standard Antiviral Drugs and Reference Inhibitors

To provide a broader perspective on the pharmacological relevance of chalcones, their antiviral activities were contextualized through a comparison with reference antiviral agents reported under comparable experimental conditions whenever available (Table 7). Overall, several chalcone derivatives exhibit inhibitory potencies within the same order of magnitude as clinically used antivirals or well-characterized reference compounds, although their performance varies considerably depending on the viral target.

In the case of flaviviruses, chalcone derivatives have demonstrated moderate but promising enzymatic inhibition. For example, the aminochalcone C9 inhibited dengue NS2B/NS3 protease with an IC_50_ of 47.9 μM, only 1.7-fold less potent than quercetin, a well-known natural flavonoid inhibitor of this target (Table 7, entry 1) [28]. Although these values remain in the micromolar range, they suggest that chalcone scaffolds can effectively engage viral proteases and may serve as viable starting points for further optimization.

More pronounced effects have been observed against influenza viruses, where several synthetic chalcone derivatives display activities comparable to, or even surpassing, that of the reference drug oseltamivir. Isoliquiritigenin-analogs showed enhanced anti-influenza A activity, with the most potent analog exhibiting more than a fourfold improvement in efficacy relative to oseltamivir while maintaining a favorable therapeutic window (Table 7, entries 2–5) [54]. Similarly, compound A9 displayed EC_50_ values comparable to oseltamivir carboxylate against H5N1 and H5N8 strains and retained activity against oseltamivir-resistant H1N1 (pdm09) isolates, highlighting the potential of chalcone scaffolds to overcome existing resistance mechanisms (Table 7, entries 6 and 7) [56].

In the context of HIV, chalcone derivatives have also demonstrated encouraging activity when compared with established inhibitors targeting different stages of the viral replication cycle. Hybrid chalcones inspired by the nevirapine scaffold showed reverse transcriptase inhibition comparable to the reference drug, with several derivatives displaying approximately twofold higher potency (Table 7, entries 8–10) [86]. In addition, chalcone-based 3-keto salicylic acid derivatives inhibited HIV integrase with IC_50_ values similar to those reported for the reference inhibitor S-1360, targeting either the strand-transfer step or both the 3′-processing and strand-transfer stages of integration (Table 7, entries 11 and 12) [87]. These findings illustrate the structural adaptability of chalcones to engage multiple viral enzymes.

In contrast, comparisons with clinically approved antivirals targeting SARS-CoV-2 reveal a more modest performance. Natural chalcones such as echinatin, licochalcone B, and xanthohumol display substantially lower potency than remdesivir, a nucleoside analog targeting the viral RNA-dependent RNA polymerase (Table 7, entries 13–15) [40,41]. Nevertheless, these compounds act through distinct mechanisms, including interference with nucleocapsid protein expression or other viral processes, suggesting that chalcone scaffolds may still represent valuable starting points for the development of antivirals with alternative mechanisms of action. It should be noted that this is the only comparison in this section in which the EC_50_ values of the chalcone and the reference drug were obtained from different studies employing distinct experimental methodologies. Therefore, this comparison should be interpreted only as a qualitative reference rather than a direct potency comparison.

A particularly notable case involves the herpes simplex virus, where licochalcone A and licochalcone B exhibited EC_50_ values comparable to the standard antiviral acyclovir (Table 7, entries 16 and 17) [44]. Importantly, licochalcone B retained activity against acyclovir-resistant HSV-2 strains, indicating that chalcones may provide viable alternatives for overcoming resistance in DNA viruses.

Altogether, these comparisons indicate that chalcone derivatives can achieve biologically relevant antiviral potencies across multiple viral targets, in some cases approaching or exceeding those of established reference compounds. However, the observed activities, as expected, are highly dependent on structural features and substitution patterns, emphasizing the importance of systematic structure–activity relationship studies. Furthermore, given that many naturally occurring chalcones display moderate cellular potency, pharmacokinetic optimization strategies, such as prodrug approaches, esterification, or modulation of lipophilicity, may represent promising avenues to enhance bioavailability and therapeutic efficacy [100]. Detailed physicochemical and pharmacokinetic analyses of the most promising chalcones are discussed in the following section.

## 10. Chalcones Against Viral Resistance Mechanisms and Global SAR Trends

Viruses, especially RNA viruses that lack proofreading mechanisms, exhibit remarkable genetic variability due to their high mutation rates, rapid replication cycles, and large population sizes [101]. Each virus possesses a characteristic viral fitness, reflecting its overall replicative capacity and ability to adapt to environmental and selective pressures, including antiviral treatment and host immune responses. However, the emergence of resistance-associated mutations is often accompanied by a fitness cost, as amino acid residue substitutions that reduce drug binding may simultaneously impair viral replication, enzymatic activity, or substrate recognition [14,102]. Consequently, resistance mutations are only likely to persist when the selective advantage conferred by reduced drug susceptibility exceeds their detrimental effects on viral fitness. Another critical determinant of resistance development is the genetic barrier, which refers to the number and nature of mutations required to substantially reduce antiviral efficacy. In general, antiviral agents targeting proteins with a high genetic barrier are less prone to resistance development, as multiple coordinated mutations are typically required before significant loss of susceptibility occurs [103]. Accordingly, highly conserved viral proteins have emerged as attractive antiviral targets because mutations occurring within these regions frequently impose substantial fitness costs, limiting the emergence of resistant variants and making them particularly suitable for the rational design of broad-spectrum antiviral agents, including chalcone-based scaffolds.

**Figure 4:**
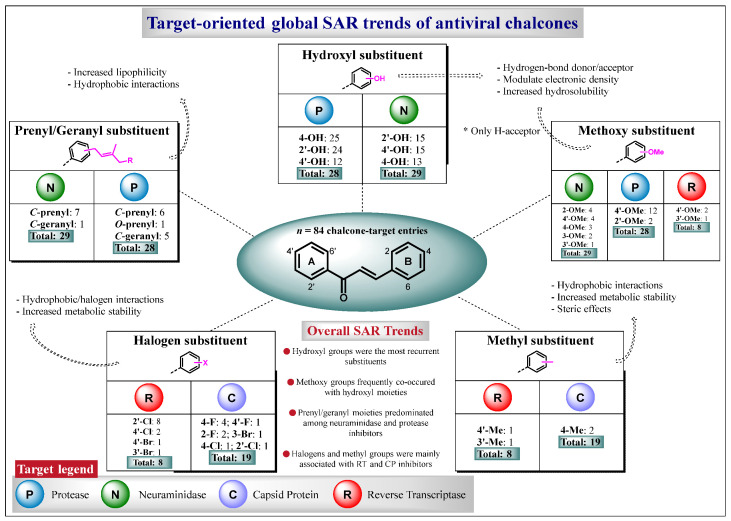
Target-oriented global SAR trends of antiviral chalcones. The figure summarizes the most recurrent structural motifs identified among the antiviral chalcones discussed in this review. Numbers indicate the frequency of each substituent at specific positions among the active chalcones reported for each target, together with the total number of active chalcones analyzed for that target. The central value (*n* = 84) represents the total number of chalcone-target entries included in the analysis. Accordingly, chalcones reported to be active against more than one viral target were counted independently for each target. Commonly reported physicochemical and medicinal chemistry contributions associated with each substituent are also highlighted. Only experimentally validated chalcones included in this review were considered for this analysis. Arrows represent the proposed contribution of each structural feature to antiviral activity, whereas dotted lines indicate recurrent substitution patterns within the chalcone scaffold. * Indicates an exception to the proposed contribution of methoxy groups.

In this context, a comprehensive analysis of the chalcones discussed in this review and their respective molecular targets provides valuable insights into the long-term therapeutic potential of chalcone-based antivirals. To facilitate this discussion, the major viral targets experimentally identified in this review were classified according to their degree of conservation and their expected genetic barrier to resistance (Table 8). However, target conservation alone does not fully predict the propensity for resistance development. The mechanism through which an antiviral interacts with its target is equally important, since compounds acting through noncompetitive or allosteric mechanisms often engage less conserved or less well-characterized regions than the catalytic site, potentially influencing the fitness costs associated with resistance mutations, and consequently, the genetic barrier to resistance [104]. Another important aspect addressed in this section is the relationship between recurrent structural motifs and specific viral targets, for which the most frequently investigated targets provided sufficient structural diversity to enable the identification of target-oriented global SAR trends (Figure 4).

Influenza virus neuraminidase (NA) was the most frequently experimentally validated molecular target identified in this review. The conservation of this enzyme across multiple influenza subtypes is not restricted to the catalytic site, known for sialic acid recognition and binding, but is also evident in several structurally constrained regions that are essential for enzymatic function [105]. This conservation may partially explain why numerous chalcones displayed comparable inhibitory activity against distinct NA subtypes, including the clinically relevant oseltamivir-resistant H274Y variant, despite predominantly acting through noncompetitive mechanisms. Nevertheless, because the precise binding sites of most chalcones remain unknown and can also vary, their long-term resistance profile is still difficult to predict. Furthermore, compensatory mutations have substantially reduced the fitness cost associated with H274Y in circulating influenza strains [102], highlighting that even highly conserved targets require continuous resistance surveillance during antiviral development.

Among the 29 chalcones reported to inhibit neuraminidase, nearly one-third displayed activity against multiple viral subtypes, suggesting that broad-spectrum inhibition may be a recurrent characteristic of this scaffold. Taking a closer look at the structural motifs of these compounds, we can identify that hydroxyl substituents represented the predominant feature, particularly at *C*-2′ and *C*-4′ (>50% frequency), followed by *C*-4 (≈45%) (Figure 4). Besides acting as hydrogen-bond donors/acceptors, hydroxyl substituents in these positions modulate the electronic properties of the conjugated enone system, both of which may favor stable interactions with amino acid residues. Methoxy and prenyl substituents were also frequently observed (≈45–50%), whereas all remaining substitution patterns appeared in fewer than 20% of the analyzed compounds. Interestingly, replacing the *C*-4′ hydroxyl group with an amino substituent preserved antiviral activity, representing a successful bioisosteric replacement that may simultaneously improve potency and refine the hydrophilic balance of future SAR optimization campaigns [54].

Viral proteases represented the second most frequently investigated target class and included enzymes from flaviviruses (serine proteases), coronaviruses (cysteine proteases), and HIV (aspartyl proteases). Despite belonging to distinct catalytic classes, these enzymes share highly conserved functional regions that are indispensable for viral polyprotein processing, making them attractive targets for broad-spectrum antiviral discovery [101,106]. Consistent with the overall trend observed throughout this review, most chalcones inhibited viral proteases through noncompetitive mechanisms, suggesting preferential interaction with regulatory or allosteric regions rather than catalytic residues. Although this strategy may reduce competition with endogenous substrates, the evolutionary conservation of these regulatory regions remains considerably less explored, making their long-term resistance profile difficult to anticipate. Particularly noteworthy is chalcone A4, which was characterized as a mixed inhibitor capable of simultaneously interacting with both the catalytic and an allosteric/regulatory site of the target protease, whereas chalcone A7 acted as a covalent competitive inhibitor [93]. This dual binding mechanism may increase the functional genetic barrier by reducing the likelihood that a single resistance mutation substantially compromises ligand binding. Moreover, the same study demonstrated minimal inhibition of four homologous host proteases, providing encouraging evidence regarding target specificity and reducing concerns about off-target toxicity [93]. Interestingly, several chalcones also displayed inhibitory activity against both SARS-CoV-2 proteases (3CL^pro^ and PL^pro^), a multitarget profile that could further reduce the probability of viral escape by simultaneously constraining independent viral functions.

Among the 28 experimentally validated protease inhibitors, hydroxyl groups again predominated, particularly at *C*-4 and *C*-2′ (>85%), reinforcing the importance of this substitution pattern across distinct antiviral targets (Figure 4). Methoxy substituents frequently co-occurred with hydroxyl groups, whereas prenyl moieties appeared in approximately 30% of the compounds. Unlike hydroxyl substituents, whose contribution has already been discussed, prenyl groups may provide an advantageous balance between lipophilicity and aqueous solubility while simultaneously promoting interactions within hydrophobic pockets, representing an attractive feature for future optimization campaigns.

Capsid or coat proteins (CPs) constituted the principal structural target identified for plant viruses in this review, particularly TMV. Although several structural domains of TMV and CMV coat proteins are highly conserved [107,108], resistance development depends not only on viral evolution, but also on host-associated selective pressures [109]. Furthermore, unlike viral enzymes, coat proteins exhibit substantial conformational flexibility and functional heterogeneity, making the precise identification of chalcone binding sites considerably more challenging.

An important methodological aspect is that several TMV studies experimentally validated direct chalcone binding through fluorescence spectroscopy, fluorescence titration, or microscale thermophoresis assays, providing stronger evidence of target engagement than docking analyses alone. As a result of extensive hybridization strategies adopted during lead optimization, robust SAR conclusions remain difficult to establish. Nevertheless, para-substituted halogens and methyl groups emerged as the most recurrent structural determinants, potentially contributing to enhanced metabolic stability, favorable steric complementarity, and hydrophobic interactions within the protein surface.

Reverse transcriptase (RT) represents one of the best-characterized antiviral targets regarding resistance evolution. Although its catalytic domains are highly conserved [110], all experimentally validated chalcones reviewed herein acted as non-nucleoside reverse transcriptase inhibitors (NNRTIs), targeting a flexible allosteric pocket historically associated with a relatively low genetic barrier. Consequently, single amino acid substitutions may confer substantial resistance while largely preserving viral replication capacity. Nevertheless, structural modifications as subtle as positional changes of halogen substituents have been shown to restore activity against established resistant RT mutants [111].

Despite the relatively limited number of compounds and studies, consistent SAR patterns were observed. Halogens at *C*-2′ (a sterically less hindered position) predominated (100%), followed by methoxy and methyl substituents at remaining positions (>37%), collectively reinforcing the importance of hydrophobic interactions for NNRTI binding.

**Table 8:** Major viral targets identified with their degree of conservation and expected genetic barrier to resistance.

Viral Target	Degree of Conservation	Expected Genetic Barrier to Resistance of Chalcones	Key Refs
Protease	High	High (catalytic site)	[101,106]
Neuraminidase	Moderate–High	Moderate–High	[105]
Capsid protein	Moderate–High	Low–Moderate	[107,109]
Reverse transcriptase	Moderate	Low (allosteric site)	[110,112]

Although the available dataset remains insufficient to establish robust SAR conclusions for several less explored viral targets, the analyses presented herein reveal important trends linking molecular target, resistance potential, and chalcone structural diversity. Collectively, these observations suggest that future optimization of chalcone-based antivirals should prioritize highly conserved viral targets while simultaneously considering the binding mechanism, since allosteric/regulatory modulation and catalytic inhibition may exhibit distinct evolutionary constraints. Furthermore, the multitarget profile displayed by several chalcones, together with the increasing interest in host-directed antiviral strategies, may further increase the functional genetic barrier by simultaneously interfering with independent viral or host pathways. Considering the limited information currently available regarding resistance selection for most chalcones, systematic evaluation of combination therapies through checkerboard or equivalent synergy assays should be encouraged, particularly against clinically relevant resistant variants. Such studies would not only identify synergistic combinations but also provide valuable insights into the long-term resistance potential, addressing one of the current barriers to the clinical translation of chalcone-based antivirals.

Finally, it should be emphasized that the SAR trends discussed herein were organized according to the reported molecular target rather than the precise binding mode. Consequently, recurrent structural features associated with a given target may encompass both competitive and noncompetitive inhibitors, which may differ substantially in their evolutionary robustness and susceptibility to resistance mutations.

## 11. In Silico Profiling of Promising Naturally Occurring Antiviral Chalcones: Physicochemical and Pharmacokinetic Considerations

To support future medicinal chemistry optimization campaigns, in silico profiling was conducted for selected naturally occurring chalcones with promising antiviral activity, in particular, compound selection was based on three main criteria: antiviral potency, experimental target validation, and breadth of antiviral spectrum. These analyses provided key insights into physicochemical properties, Absorption, Distribution, Metabolism, Excretion, and Toxicity (ADMET) parameters, and drug-likeness descriptors (factors that are strongly associated with overall pharmacokinetic and pharmacodynamic performance). Early-stage in silico assessments are widely recognized as effective tools for prioritizing promising scaffolds and reducing the risk of downstream failures commonly linked to poor aqueous solubility, limited membrane permeability, or rapid metabolic clearance [113,114]. It is important to emphasize that although in silico evaluations can support early-stage medicinal chemistry decision-making, they do not replace experimentally determined pharmacokinetic, toxicological, or biochemical data.

Predicted ADMET-related parameters were obtained using the SwissADME [115] and ADMETlab 3.0 [116] web servers, both accessed December 2025. Canonical SMILES strings used as input are provided in the Supplementary Materials (Table S2). Unless otherwise stated, all predicted ADMET-related parameters were interpreted according to the default decision thresholds and categorical classifications implemented by SwissADME and ADMETlab 3.0. Molecular weight (MW), molar refractivity (MR), topological polar surface area (TPSA), numbers of hydrogen-bond donors and acceptors, rotatable bonds, lipophilicity estimated as consensus log*P*, and water solubility estimated by the ESOL model (log*S* and solubility class), as well as compliance with Lipinski’s and Veber’s criteria, are summarized in Table 9. ADMET parameters, including human colorectal adenocarcinoma (Caco-2) permeability, Madin–Darby canine kidney (MDCK) permeability, skin permeability (log*K*_p_), plasma protein binding (PPB), cytochrome P450 isoforms 1A2 and 2C9 (CYP1A2 and CYP2C9, respectively), plasma clearance (CL), elimination half-life (t_1/2_), and predicted A549 cytotoxicity and rat oral acute toxicity, are presented in Table 10. In addition, the BOILED-Egg model predicting human intestinal absorption (HIA) and blood–brain barrier (BBB) permeability is illustrated in Figure 4.

Among the most widely accepted criteria for assessing drug-likeness are Lipinski’s rule of five and Veber’s rules. Lipinski’s rule defines threshold values commonly observed in orally active drugs, including molecular weight below 500 g/mol, fewer than 5 hydrogen-bond donors, fewer than 10 hydrogen-bond acceptors, and a calculated log*P* below 5 [117]. Complementarily, Veber’s rules associate good oral bioavailability in rat models with 10 or fewer rotatable bonds and a TPSA not exceeding 140 Å^2^ [118]. In this context, the physicochemical properties of all evaluated chalcones were consistent with these criteria. Molecular weights ranged from 256.25 to 438.51 g/mol, molar refractivity values from 72.32 to 128.11 cm^3^/mol, TPSA values from 66.76 to 107.22 Å^2^, hydrogen-bond donor/acceptor counts from 2 to 6, and rotatable bonds from 3 to 10, supporting their favorable drug-likeness profiles.

The consensus log*P* values of the compounds ranged from 2.37 to 5.50, while ESOL log*S* values ranged from −6.66 to −3.41, highlighting the pronounced impact of prenyl substitution on increasing lipophilicity while concomitantly reducing aqueous solubility. The appropriate balance between these parameters is critical during in vivo and clinical development, as it directly influences bioavailability, membrane permeability, and systemic exposure. Such properties are commonly modulated through medicinal chemistry strategies, including prodrug design and targeted structural modifications [119].

**Figure 5:**
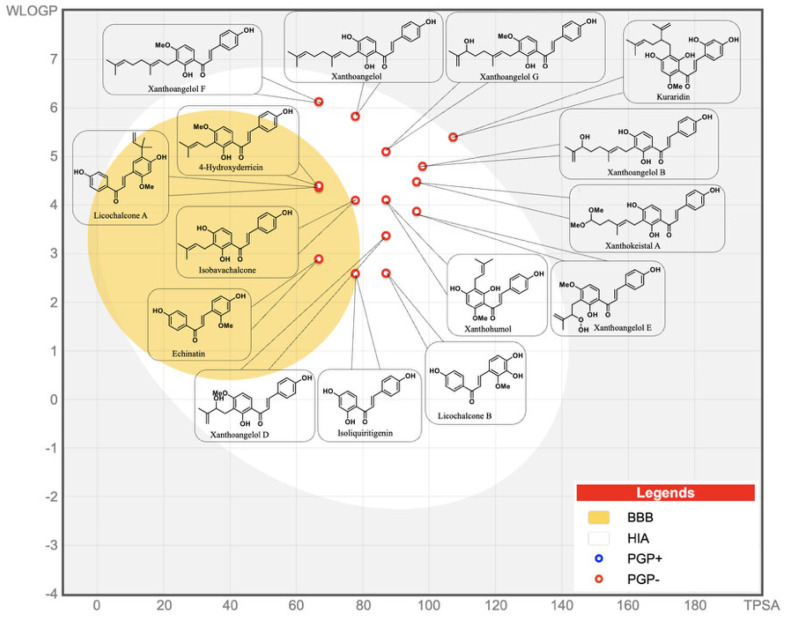
BOILED-Egg model predicting human intestinal absorption (HIA) and blood–brain barrier (BBB) permeability. Compounds located in the white zone are predicted to have a high probability of passive gastrointestinal absorption, whereas those in the yellow zone are predicted to readily penetrate the brain. Dot colors indicate compound interaction with P-glycoprotein (P-gp): blue denotes predicted P-gp substrates (PGP+), and red denotes predicted non-substrates (PGP−).

Regarding absorption, the BOILED-Egg model predicted that all compounds have a high probability of passive intestinal absorption and are non-substrates of P-glycoprotein (P-gp), a membrane efflux transporter commonly associated with reduced intracellular drug accumulation (Figure 5). Consistently, the Caco-2 cell model predicted favorable permeability for most compounds, with the exception of xanthokeistal A, supporting an overall good potential for oral absorption. In contrast, permeability predictions based on the MDCK model indicated low passive permeability for all compounds. This apparent discrepancy likely reflects intrinsic differences between the two cellular models, as MDCK cells are more sensitive to lipophilicity, efflux-related mechanisms, and variations in transporter expression profiles [120]. Skin permeability, expressed as log*K*_p_ values, ranged from −6.20 to −3.60, indicating limited to moderate dermal permeation. Although no strict universal thresholds are defined for log*K*_p_, values within this range are commonly associated with low systemic exposure following dermal administration, which is consistent with compounds primarily intended for oral administration [121].

The BOILED-Egg model also predicted that licochalcone A, echinatin, 4-hydroxyderricin, and isoliquiritigenin are capable of crossing the blood–brain barrier (BBB) (Figure 5). This property may be particularly relevant for these compounds, as they have been reported to target neuroinvasive viruses, including H1N1, SARS-CoV-2, EV-A71, HSV-2, and ZIKV [122,123,124]. Plasma protein binding (PPB), a key determinant of drug distribution, was predicted to be high for all compounds, with values exceeding the commonly accepted optimal threshold of 90%. While high PPB may prolong systemic exposure by acting as a circulating drug reservoir, it can also reduce the free, pharmacologically active fraction, potentially impacting therapeutic efficacy and dosing requirements [125].

From a metabolic perspective, most compounds were predicted to inhibit cytochrome P450 (CYP) isoforms 1A2 and 2C9, heme-containing enzymes that play central roles in phase I xenobiotic and drug metabolism. Although CYP inhibition may raise concerns regarding potential drug–drug interactions during clinical development, it can also contribute to reduced metabolic clearance and extended elimination half-life [126,127].

Excretion-related parameters revealed low to moderate plasma clearance (CL) values and elimination half-lives (t_1/2_) ranging from 0.842 to 1.500 h, suggesting moderate metabolic turnover. Finally, toxicity predictions based on A549 cytotoxicity and rat oral acute toxicity models indicated a moderate risk of being cytotoxic or to exhibit acute toxicity (LD_50_ < 500 mg/kg), with estimated probabilities ranging from 7.4% to 47.3% and 9.9% to 42.7%, respectively. Overall, these in silico results highlight a balance between favorable pharmacokinetic properties and potential liabilities, underscoring the need for further experimental validation and structure-guided optimization.

## 12. Conclusions

Our review summarized and critically discussed the main advances regarding natural and synthetic chalcones with antiviral activity, emphasizing their molecular targets, mechanisms of action, pharmacological potential compared to standard agents, and in silico ADMET profiling. Collectively, the evidence underscores chalcones as a versatile structural scaffold capable of modulating multiple stages of the viral life cycle, including the direct inhibition of viral proteases and polymerases, interference with viral entry and replication, and modulation of host-related responses. Notably, several chalcones have demonstrated potency values comparable to reference antiviral drugs, particularly against influenza A, HIV, and HSV, reinforcing their potential as promising lead compounds. Beyond summarizing the antiviral activity, this review provides a comprehensive discussion of the major viral targets from a resistance perspective, integrating target conservation, expected genetic barriers, and global SAR trends. These observations may assist future medicinal chemistry campaigns by highlighting structural features associated with conserved antiviral targets while emphasizing the importance of experimentally evaluating resistance development and synergistic combinations to improve the long-term therapeutic potential of chalcone-based antivirals. In addition, complementary in silico ADMET profiling provided a pharmacokinetic-oriented perspective on the most promising naturally occurring chalcones. These analyses revealed generally favorable drug-likeness profiles, intestinal absorption potential, and manageable toxicity risks while also identifying key liabilities related to solubility, plasma protein binding, and cytochrome P450 inhibition. Such findings emphasize the importance of early pharmacokinetic considerations to guide rational structure optimization and reduce attrition during later stages of drug development.

Overall, chalcones have been reported to target a broad range of viral enzymes and structural proteins across multiple virus families, including RNA and DNA viruses of human, animal, and plant origin. This diversity of targets and viral systems highlights the adaptability of the chalcone scaffold for antiviral drug discovery. However, despite extensive in silico targeting predictions and in vitro investigations, relatively few or no chalcone derivatives have advanced to in vivo validation or detailed pharmacokinetic evaluation, which continues to limit their translational progression. Future research efforts should therefore prioritize the integration of medicinal chemistry, molecular biology, and pharmacokinetic strategies to: (i) perform deeper mechanistic studies clarifying specific modes of action; (ii) conduct rational structural optimization to improve pharmacokinetic properties and metabolic stability; and (iii) explore combination therapies with approved antivirals to expand antiviral coverage and mitigate resistance. In summary, chalcones remain a pharmacologically valuable class of compounds, and the consolidation of rational design strategies together with robust experimental validation may establish them as strong candidates for the next generation of broad-spectrum antiviral agents.

## Figures and Tables

**Table 3:** Anti-influenza A activity of synthetic chalcones.

S/N Chalcones	Structure	Virus	Antiviral Activity	Target/Mode of Action	Ref
34—2′,4′-dihydroxy-4-methoxy chalcone	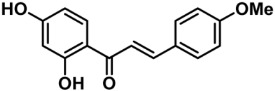	Influenza A	IC_50_ = 2.23 µM (H1N1 NA)	Neuraminidase (noncompetitive)	[53]
35—2′,4′-dihydroxy-3-methoxy chalcone	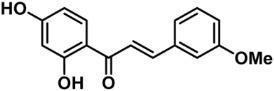	Influenza A	IC_50_ = 8.71 µM (H1N1 NA)	Neuraminidase (noncompetitive)	[53]
36—2′,4′-dihydroxy-3-chloro chalcone	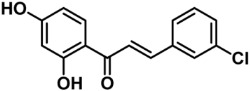	Influenza A	IC_50_ = 3.58 µM (H1N1 NA)	Neuraminidase (noncompetitive)	[53]
37—2′,4′-dihydroxy-4-nitro chalcone	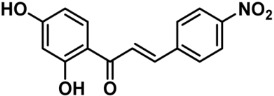	Influenza A	IC_50_ = 6.64 µM (H1N1 NA)	Neuraminidase (noncompetitive)	[53]
38—1a (R = 2-Cl)	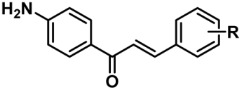	Influenza A	EC_50_ = 2.36 nM/SI: 72,288 IC_50_ = 6.64 µM (H1N1 NA)	Neuraminidase (noncompetitive)	[54]
39—1b (R = 3-Cl)		Influenza A	EC_50_ = 2.36 nM/SI: 63,729 IC_50_ = 5.56 µM (H1N1 NA)	Neuraminidase (noncompetitive)	[54]
40—1e (R = 3-OMe)		Influenza A	EC_50_ = 1.71 nM/SI: 105,497 IC_50_ = 4.10 µM (H1N1 NA)	Neuraminidase (noncompetitive)	[54]
41—1f (R = 4-OMe)		Influenza A	EC_50_ = 2.76 nM/SI: 72,428 IC_50_ = 3.58 µM (H1N1 NA)	Neuraminidase (noncompetitive)	[54]
42—1h (R = 3-NO2)		Influenza A	EC_50_ = 5.26 nM/SI: 24,316 IC_50_ = 8.26 µM (H1N1 NA)	Neuraminidase (noncompetitive)	[54]
43—1i (R = 4-NO2)		Influenza A	EC_50_ = 4.62 nM/SI: 24,372 IC_50_ = 7.86 µM (H1N1 NA)	Neuraminidase (noncompetitive)	[54]
44—2c (R = 4-Cl)	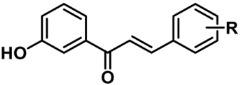	Influenza A	EC_50_ = 15.64 nM/SI: 7417 IC_50_ = 12.36 µM (H1N1 NA)	Neuraminidase (noncompetitive)	[54]
45—2f (R = 4-OMe)		Influenza A	EC_50_ = 8.62 nM/SI: 33,921 IC_50_ = 5.25 µM (H1N1 NA)	Neuraminidase (noncompetitive)	[54]
46—2i (R = 4-NO2)		Influenza A	EC_50_ = 12.65 nM/SI: 13,423 IC_50_ = 8.65 µM (H1N1 NA)	Neuraminidase (noncompetitive)	[54]
47—1c-H	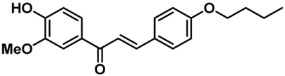	Influenza A	IC_50_ = 27.63–28.11 µM (H1N1 and H5N1 NAs)	Neuraminidase (noncompetitive)	[55]
48—2b-H	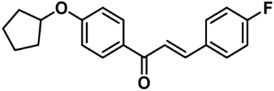	Influenza A	IC_50_ = 73.17–87.54 µM (H1N1 and H5N1 NAs)	Neuraminidase (noncompetitive)	[55]
49—A9	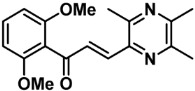	Influenza A	EC_50_ = 7.34 µM/SI: 30.9	Not reported	[56]

IC_50_ = half-maximal inhibitory concentration; EC_50_ = half-maximal effective concentration; SI = selectivity index (CC_50_/IC_50_).

**Table 4:** Anti-TMV/CMV activity of synthetic chalcones.

S/N Chalcones	Structure	Virus	Antiviral Activity	Target/Mode of Action	Ref
50—3h	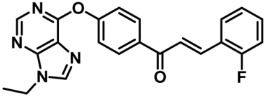	TMV	EC_50_ = 407.9 µg/mL *K*_d_ = 6.7 mM (TMV-CP)	TMV-CP	[60]
51—3o	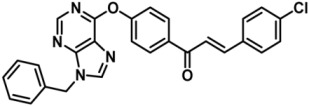	TMV	EC_50_ = 301.1 µg/mL *K*_d_ = 5.1 µM (TMV-CP)	TMV-CP	[60]
52—3s	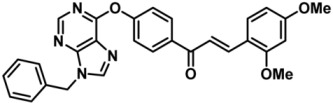	TMV	EC_50_ = 315.7 µg/mL *K*_d_ = 155 µM (TMV-CP)	TMV-CP	[60]
53—3w	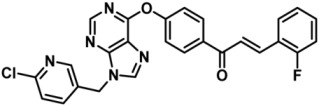	TMV	EC_50_ = 282.3 µg/mL	Not reported	[60]
54—3x	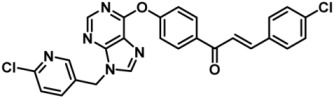	TMV	EC_50_ = 230.5 µg/mL	Not reported	[60]
55—3n′	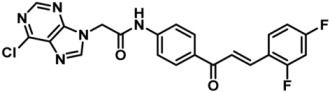	TMV	EC_50_ = 452 µg/mL *K*_d_ = 79.8 µM (TMV-CP)	TMV-CP	[70]
56—3p′	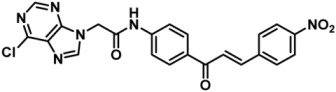	TMV	EC_50_ = 439 µg/mL	Not reported	[70]
57—d2	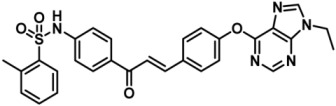	TMV	EC_50_ = 51.7 µg/mL *K*_d_ = 12.16 µM (TMV-CP)	TMV-CP	[61]
58—d7	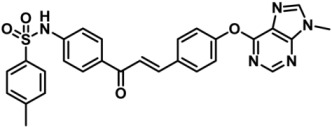	TMV	EC_50_ = 53.5 µg/mL *K*_d_ = 20.63 µM (TMV-CP)	TMV-CP	[61]
59—7g	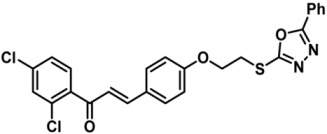	TMV	EC_50_ = 33.66 µg/mL *K*_d_ = 5.93 µM (TMV-CP)	TMV-CP	[59]
60—7l	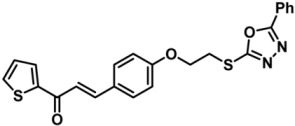	TMV	EC_50_ = 33.97 µg/mL *K*_d_ = 6.15 µM (TMV-CP)	TMV-CP	[59]
61—8h	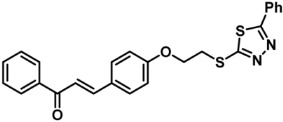	TMV	EC_50_ = 33.87 µg/mL *K*_d_ = 6.02 µM (TMV-CP)	TMV-CP	[59]
62—8l	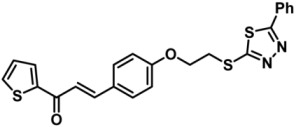	TMV	EC_50_ = 30.57 µg/mL *K*_d_ = 5.04 µM (TMV-CP)	TMV-CP	[59]
63—5l	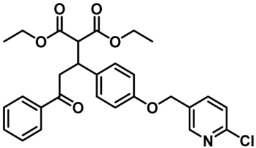	CMV	EC_50_ = 186.17 µg/mL	Not reported	[69]
64—5n	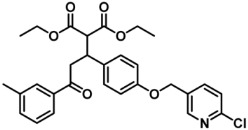	CMV	EC_50_ = 211.47 µg/mL	Not reported	[69]
65—H9	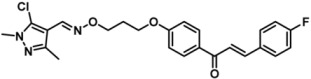	TMV	EC_50_ = 166.9 µg/mL *K*_d_ = 0.0096 ± 0.0045 μM (TMV-CP)	TMV-CP	[62]
66—N22	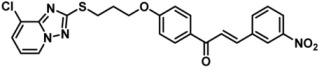	TMV	EC_50_ = 77.64 µg/mL *K*_d_ = 0.0076 ± 0.0007 μM (TMV-CP)	TMV-CP	[63]
67—S14	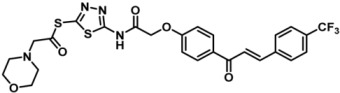	TMV	EC_50_ = 91.8 µg/mL *K*_d_ = 0.0126 ± 0.0058 μM (TMV-CP)	TMV-CP	[64]
68—S7	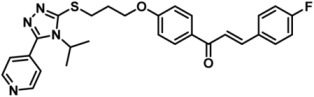	TMV	EC_50_ = 89.7 µg/mL *K*_d_ = 0.5340 ± 0.2233 μM (TMV-CP)	TMV-CP	[71]
69—T19	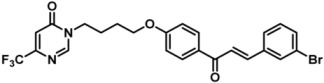	TMV	EC_50_ = 234.9 µg/mL *K*_d_ = 0.0031 ± 0.0009 μM (TMV-CP)	TMV-CP	[65]
70—D11	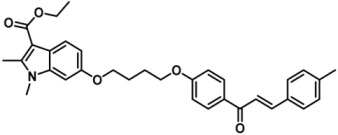	TMV	EC50 = 107.4 µg/mL *K*_d_ = 0.0030 ± 0.0014 µM (TMV-CP)	TMV-CP	[66]
71—Z15	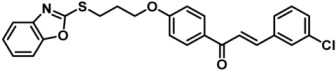	TMV	EC50 = 101.97 µg/mL	Not reported	[67]
72—B3	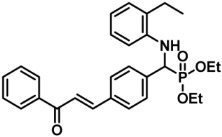	TMV	EC_50_ = 356.7 µg/mL *K*_a_ = 2.51 × 10^8^ M^−1^ (TMV-CP)	TMV-CP	[68]
73—4	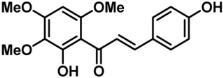	TMV	EC_50_ = 52.1 µM	Not reported	[75]
74—L1	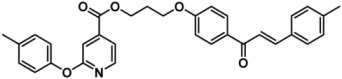	TMV	EC_50_ = 140.5 µg/mL	Not reported	[72]
75—L4	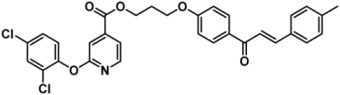	TMV	EC_50_ = 90.7 µg/mL *K*_d_ = 0.00149 ± 0.00071 μM (TMV-CP)	TMV-CP	[72]

IC_50_ = half-maximal inhibitory concentration; EC_50_ = half-maximal effective concentration; SI = selectivity index (CC_50_/IC_50_); *K*_a_ = association constant; *K*_d_ = dissociation constant.

**Table 5:** Anti-HIV activity of synthetic chalcones.

S/N Chalcones	Structure	Virus	Antiviral Activity	Target/Mode of Action	Ref
76—15	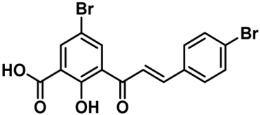	HIV-1	EC_50_ = 8.7 µM/SI = 2.8 IC_50_ = 11 ± 4 (HIV IN—3′-processing) IC_50_ = 5 ± 2 (HIV IN—strand transfer)	Integrase	[87]
77—25	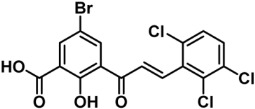	HIV-1	EC_50_ = 7.3 µM/SI = 3.1 IC_50_ = < 3.7 (HIV IN—strand transfer)	Integrase	[87]
78—A1	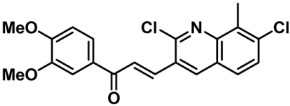	HIV-1	EC_50_ = 5.78 µM/SI = 6.9 IC_50_ = 0.15 µg/mL (HIV RT) *K*_i_ = 104.97 µM (HIV RT)	Non-nucleoside reverse transcriptase	[86]
79—A4 (R = Br)	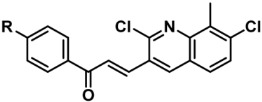	HIV-1	EC_50_ = 1.43 µM/SI = 22.4 IC_50_ = 0.10 µg/mL (HIV RT) *K*_i_ = 413.39 µM (HIV RT)	Non-nucleoside reverse transcriptase	[86]
80—A6 (R = Cl)		HIV-1	EC_50_ = 1.58 µM/SI = 14.5 IC_50_ = 0.11 µg/mL (HIV RT) *K*_i_ = 306.22 µM (HIV RT)	Non-nucleoside reverse transcriptase	[86]
81—A7 (R = OMe)		HIV-1	EC_50_ = 7.78 µM/SI = 6.4 IC_50_ = 0.25 µg/mL (HIV RT) *K*_i_ = 791.79 µM (HIV RT)	Non-nucleoside reverse transcriptase	[86]
82—A8	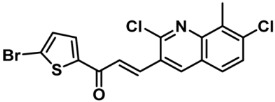	HIV-1	EC_50_ = 1.98 µM/SI = 13.6 IC_50_ = 0.14 µg/mL (HIV RT) *K*_i_ = 418.46 µM (HIV RT)	Non-nucleoside reverse transcriptase	[86]
83—A10	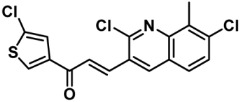	HIV-1	EC_50_ = 1.89 µM/SI = 13.1 IC_50_ = 0.13 µg/mL (HIV RT) *K*_i_ = 371.48 µM (HIV RT)	Non-nucleoside reverse transcriptase	[86]
84—A12	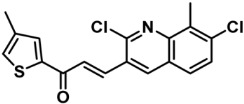	HIV-1	EC_50_ = 7.90 µM/SI = 7.1 IC_50_ = 0.19 µg/mL (HIV RT) *K*_i_ = 210.60 µM (HIV RT)	Non-nucleoside reverse transcriptase	[86]
85—A13	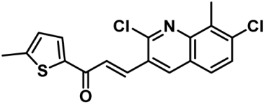	HIV-1	EC_50_ = 8.12 µM/SI = 6.4 IC_50_ = 0.18 µg/mL (HIV RT) *K*_i_ = 1.10 µM (HIV RT)	Non-nucleoside reverse transcriptase	[86]
86—5h	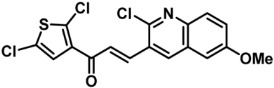	HIV-1	EC_50_ = 1.1 µM/SI = 2.4	Not reported	[88]

IC_50_ = half-maximal inhibitory concentration; EC_50_ = half-maximal effective concentration; SI = selectivity index (CC_50_/IC_50_); *K*_i_ = inhibition constant.

**Table 7:** Comparison of the inhibitory concentration of chalcones and standard inhibitors/antiviral drugs.

S/N	Chalcones	Inhibitory Concentration	Standard Drug	Inhibitory Concentration	Virus	Refs
1	C9	NS2B-NS3^pro^: IC_50_ = 47.91 µM	Quercetin	NS2-NS3^pro^: IC_50_ = 28.73 µM	DENV	[28]
2	1e	EC_50_ = 1.71 nM	Oseltamivir	EC_50_ = 7.1 nM	H1N1	[54]
3	1a	EC_50_ = 2.36 nM			H1N1	[54]
4	1b	EC_50_ = 2.36 nM			H1N1	[54]
5	1f	EC_50_ = 2.76 nM			H1N1	[54]
6	A9	EC_50_ = 7.34 µM	Oseltamivir carboxylate	EC_50_ = 5.70 µM	H5N1	[56]
7	A9	EC_50_ = 10.85 µM	Oseltamivir carboxylate	EC_50_ = 6.49 µM	H5N8	[56]
8	A4	RT: IC_50_ = 0.10 µg/mL	Nevirapine	RT: IC_50_ = 0.23 µg/mL	HIV	[86]
9	A6	RT: IC_50_ = 0.11 µg/mL			HIV	[86]
10	A10	RT: IC_50_ = 0.13 µg/mL			HIV	[86]
11	15	3′-P: IC_50_ = 11.0 µM ST: IC_50_ = 5.0 µM	S-1360	3′-P: IC_50_ = 11.0 µM ST: IC_50_ = 0.6 µM	HIV	[87]
12	25	ST: IC_50_ = <3.7 µM			HIV	[87]
13	Echinatin	EC_50_ = 7.862 µM	Remdesivir	EC_50_ = 37 nM	SARS-CoV-2	[41,95]
14	Licochalcone B	EC_50_ = 15.53 µM			SARS-CoV-2	[41,95]
15	Xanthohumol	EC_50_ = 3.3 µM			SARS-CoV-2	[40,95]
16	Licochalcone A	EC_50_ = 1.73 µM	Acyclovir	EC_50_ = 0.51 µM	HSV-2	[44]
17	Licochalcone B	EC_50_ = 3.80 µM			HSV-2	[44]

IC_50_ = half-maximal inhibitory concentration; EC_50_ = half-maximal effective concentration.

**Table 9:** Predicted physicochemical properties, lipophilicity, water solubility, and drug-likeness descriptor for selected naturally occurring chalcones.

S/N	Chalcones	Physicochemical Properties						Lipophilicity	Water Solubility		Drug-Likeness	
		MW (g/mol)	MR (cm^3^/mol)	TPSA (Å^2^)	Num. H-Bond Acceptors	Num. H-Bond Donors	Num. Rotatable Bonds	cLog*P*	ESOL log*S*	ESOL Class	Lipinski	Veber
**1**	Isoliquiritigenin	256.25	72.32	77.76	4	3	3	2.37	−3.70	Soluble	Yes; 0	Yes
**2**	Isobavachalcone	324.37	96.04	77.76	4	3	5	3.83	−5.10	Moderately soluble	Yes; 0	Yes
**3**	Xanthohumol	354.40	102.53	86.99	5	3	6	3.76	−5.18	Moderately soluble	Yes; 0	Yes
**4**	Echinatin	270.28	76.79	66.76	4	2	4	2.55	−3.55	Soluble	Yes; 0	Yes
**5**	Licochalcone A	338.40	100.39	66.76	4	2	6	3.93	−4.98	Moderately soluble	Yes; 0	Yes
**6**	Licochalcone B	286.28	78.81	86.99	5	3	4	2.14	−3.41	Soluble	Yes; 0	Yes
**7**	4-Hydroxyderricin	338.40	100.51	66.76	4	2	6	4.19	−5.32	Moderately soluble	Yes; 0	Yes
**8**	Xanthoangelol	392.49	119.60	77.76	4	3	8	5.30	−6.44	Poorly soluble	Yes; 0	Yes
**9**	Xanthoangelol B	408.49	120.76	97.99	5	4	9	4.48	−5.84	Moderately soluble	Yes; 0	Yes
**10**	Xanthoangelol D	354.40	101.67	86.99	5	3	7	3.35	−4.68	Moderately soluble	Yes; 0	Yes
**11**	Xanthoangelol E	370.40	103.16	96.22	6	3	8	3.44	−4.73	Moderately soluble	Yes; 0	Yes
**12**	Xanthoangelol F	406.51	124.07	66.76	4	2	9	5.50	−6.66	Poorly soluble	Yes; 0	Yes
**13**	Xanthoangelol G	422.51	125.23	86.99	5	3	10	4.87	−6.06	Poorly soluble	Yes; 0	Yes
**14**	Xanthokeistal A	412.48	117.44	96.22	6	3	10	4.10	−5.32	Moderately soluble	Yes; 0	Yes
**15**	Kuraridin	438.51	128.11	107.22	6	4	9	4.78	−6.36	Poorly soluble	Yes; 0	Yes

Prediction of physicochemical properties, lipophilicity, water solubility, and drug-likeness descriptors were performed using publicly available tools (SwissADME and ADMETlab 3.0), providing a comparative overview of antiviral chalcones. MW: molecular weight; MR: molar refraction; TPSA: topological polar surface area; cLog*P*: consensus log *P*; ESOL: estimated solubility; Lipinski’s Rule of Five: Rule acceptance and number of violations; Veber = Rule acceptance.

**Table 10:** Predicted ADMET parameters for selected naturally occurring chalcones.

S/N	Chalcones	Absorption			Distribution	Metabolism		Excretion		Toxicity	
		Caco-2 Permeability (log)	MDCK Permeability (cm/s)	Skin Permeability (log *K*_p_)	PPB (%)	CYP1A2 Inhibitor	CYP2C9 Inhibitor	CL Plasma (mL/min/kg)	t_1/2_ (h)	A549 Toxicity (%)	Rat Oral Acute Toxicity (%)
**1**	Isoliquiritigenin	−4.839	−4.798	−5.61	96.1	Yes	Yes	5.761	1.376	23.9	10.5
**2**	Isobavachalcone	−5.045	−4.797	−4.66	95.4	Yes	Yes	5.531	1.121	47.5	33.6
**3**	Xanthohumol	−5.056	−4.821	−4.86	95.6	Yes	Yes	7.297	0.865	40.4	42.7
**4**	Echinatin	−4.831	−4.802	−5.85	94.5	Yes	Yes	12.156	1.298	13.5	13.6
**5**	Licochalcone A	−5.023	−4.856	−4.89	97.7	Yes	Yes	10.576	1.088	45.4	35.8
**6**	Licochalcone B	−5.010	−4.823	−6.20	96.1	Yes	Yes	14.556	1.500	36.4	15.4
**7**	4-Hydroxyderricin	−5.039	−4.797	−4.51	96.7	Yes	Yes	9.911	0.877	35.8	31.0
**8**	Xanthoangelol	−5.083	−4.810	−3.75	97.5	Yes	Yes	4.601	1.126	47.3	22.2
**9**	Xanthoangelol B	−5.139	−4.808	−4.55	95.8	Yes	Yes	4.752	1.182	42.5	20.3
**10**	Xanthoangelol D	−5.084	−4.798	−5.35	96.1	Yes	Yes	10.393	1.026	7.4	9.9
**11**	Xanthoangelol E	−5.083	−4.803	−5.41	96.9	No	Yes	8.72	1.154	8.6	17.9
**12**	Xanthoangelol F	−5.042	−4.772	−3.60	98.2	Yes	Yes	9.505	0.842	42.7	23.4
**13**	Xanthoangelol G	−5.12	−4.770	−4.40	97.6	Yes	Yes	9.474	0.894	35.9	21.2
**14**	Xanthokeistal A	−5.315	−4.895	−5.11	95.3	No	Yes	4.97	1.125	28.5	19.5
**15**	Kuraridin	−5.098	−4.808	−4.33	96.9	No	Yes	2.258	1.483	42.2	37.9

Prediction of ADMET parameters was performed using publicly available tools (SwissADME and ADMETlab 3.0), providing a comparative overview to support pharmacokinetic-oriented prioritization of antiviral chalcones. Tools’ internal thresholds = Caco-2 permeability: optimal value > −5.15 (log units). MDCK permeability: low (<2 × 10^−6^ cm/s), medium (2–20 × 10^−6^ cm/s), and high (>20 × 10^−6^ cm/s). Skin permeability is expressed as log *K*_p_, where *K*_p_ represents the skin permeability coefficient (cm/s). PPB: plasma protein binding, with optimal values < 90%. CYP1A2 and CYP2C9 refer to cytochrome P450 isoforms 1A2 and 2C9, respectively. Plasma clearance (CL): high (>15 mL/min/kg), moderate (5–15 mL/min/kg), and low (<5 mL/min/kg). T_1/2_: elimination half-life. A549 toxicity and rat oral acute toxicity represent the predicted probability of cytotoxicity.

## Data Availability

Data are contained within the article and Supplementary Information.

## Supplementary Materials

The following supporting information can be downloaded at: https://www.mdpi.com/article/10.3390/v18070806/s1, Table S1: Antiviral activity of chalcones; Table S2: SMILES strings used for in silico ADMET profiling.
