# Supplementary material for: Chalcones as a Versatile Antiviral Scaffold: Molecular Targets, ADMET Profiles, and Translational Challenges
Source: Viruses. 2026 Jul 22;18(7):806. doi: 10.3390/v18070806 (PMC13431542; doi:10.3390/v18070806)
Supplement: Supplementary file 1 [file viruses-18-00806-s001.zip › viruses-4392985-supplementary.pdf]

Supplementary Information

Chalcones as a Versatile Antiviral Scaffold: Molecular Targets, ADMET Profiles, and Translational Challenges

Alvaro Luiz Helena, Patrick Rômbola Ozanique, Kevin Henrique Souza Lima, Wellington Negri Tondato,
Victor Yukio Ichikawa Baio, Otávio Henrique Locateli Soares and Luis Octávio Regasini *

Department of Chemistry and Environmental Sciences, Institute of Biosciences, Humanities and Exact Sciences,
São Paulo State University (UNESP), São José do Rio Preto 15054-000, SP, Brazil;
alvaro.helena@unesp.br (A.L.H.);
patrick.ozanique@unesp.br (P.R.O.); kevin.lima@unesp.br (K.H.S.L.);
w.tondato@unesp.br (W.N.T.);
victor.baio@unesp.br (V.Y.I.B.); otavio.henrique@unesp.br (O.H.L.S.)
* Correspondence: luis.regasini@unesp.br

Table S1. Antiviral activity of chalcones.

S/N	Chalcones	Structure	Virus	Study Model	Inhibitory Concentration IC_{50}/EC_{50} Docking energy/score Selectivity/Therapeutic index SI/TI	Cytotoxic Concentration CC_{50}	Inhibition Stage	Target / Mode of action	Ref
**1**	Cardamonin	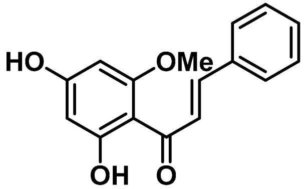	DENV2	*In vitro*	NS3: *Ki* = 377 ± 77 µM (noncompetitive) Percentage inhibition NS2B/NS3 (120 ppm) = 39.4%; (240 ppm) = 50.1%; (400 ppm) = 71.3%	Not reported	Viral replication	Noncompetitive NS2B/NS3 protease complex inhibitor	[29]
			HCoV-OC43	*In vitro*	HCoV-OC43/MRC-5 cell: IC_{50} = 3.62 µM /SI = >13.81	MRC-5 cell: CC_{50} = >50 µM	Viral replication	p38 MAPK signaling pathway activator	[30]
**2**	SC11	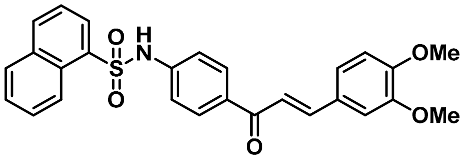	DENV2	*In vitro*	DENV2/Vero: EC_{50} = 3.13 ± 0.52 µM /SI = 10.67	Vero cell: CC_{50} = 33.90 ± 1.63 µM	During and post-infection	Not reported	[30]
**3**	SC20	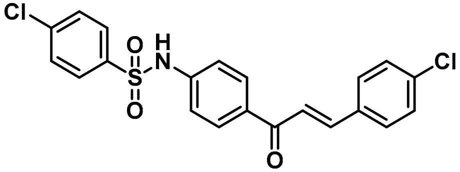	DENV2	*In vitro*	DENV2/Vero: EC_{50} = 2.21 ± 0.56 µM /SI = 7.58	Vero cell: CC_{50} = 16.77 ± 1.88 µM	During and post-infection	Not reported	[30]
**4**	SC22	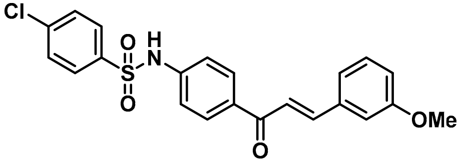	DENV2 DENV1 DENV3 DENV4	*In vitro*	DENV2/Vero: EC_{50} = 0.71 ± 0.07 µM /SI = 20.60 DENV2/LLC/MK2: EC_{50} = 1.83 ± 0.49 µM /SI = 38.51 DENV2/A549: EC_{50} = 8.63 ± 0.19 µM /SI = 5.96 DENV2/HepG2: EC_{50} = 5.26 ± 0.21 µM /SI = 10.11 DENV1/Vero: EC_{50} = 0.78 ± 0.18 µM /SI = 18.75 DENV3/Vero: EC_{50} = 0.94 ± 0.17 µM /SI = 15.56 DENV4/Vero: EC_{50} = 0.77 ± 0.24 µM /SI = 19 NS5 MTase: IC_{50} = 16.85 ± 2.15 µM	Vero cell: CC_{50} = 14.63 ± 1.65 µM LLC/MK2 cell: CC_{50} = 70.48 ± 4.43 µM A549 cell: CC_{50} = 51.42 ± 3.73 µM HepG2 cell: CC_{50} = 54.21 ± 3.02 µM	During and post-infection	Methyltransferase inhibitor as partial target	[30]
**5**	SC24	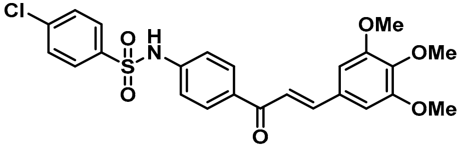	DENV2	*In vitro*	DENV2/Vero: EC_{50} = 0.65 ± 0.15 µM /SI = 21.11	Vero cell: CC_{50} = 13.72 ± 0.28 µM	During and post-infection	Not reported	[30]

S/ N	Chalcones	Structure	Virus	Study Model	Inhibitory Concentration IC_{50}/EC_{50} Docking energy/score Selectivity/Therapeutic index SI/TI	Cytotoxic Concentration CC_{50}	Inhibition Stage	Target / Mode of action	Ref
**6**	SC27	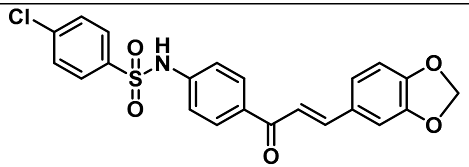	DENV2 DENV1 DENV3 DENV4	*In vitro*	DENV2/Vero: EC_{50} = 3.18 ± 0.89 µM /SI = 9.75 DENV2/LLC/MK2: EC_{50} = 2.39 ± 0.16 µM /SI = >41.84 DENV2/A549: EC_{50} = 3.33 ± 0.57 µM /SI = 30.03 DENV2/HepG2: EC_{50} = 4.80 ± 0.07 µM /SI = >20.83 DENV1/Vero: EC_{50} = 4.46 ± 0.43 µM /SI = 6.96 DENV3/Vero: EC_{50} = 3.15 ± 0.78 µM /SI = 9.85 DENV4/Vero: EC_{50} = 4.44 ± 1.65 µM /SI = 6.98 NS5 MTase: IC_{50} = 11.58 ± 2.06 µM	Vero cell: CC_{50} = 31.02 ± 1.75 µM LLC/MK2 cell: CC_{50} = >100 µM A549 cell: CC_{50} = 51.42 ± >100 µM HepG2 cell: CC_{50} = 54.21 >100 µM	During and post-infection	Methyltransferase inhibitor	[30]
**7**	C9	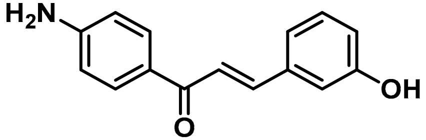	DENV2	*In silico* *In vitro*	NS2B/NS3: IC_{50} = 47.9 µM	Not reported	Viral replication	NS2B/NS3 protease complex inhibitor	[28]
**8**	Sofalcone	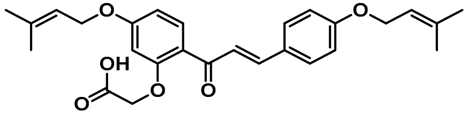	DENV2 DENV1 DENV3 DENV4	*In vitro* *In vivo*	DENV2/Huh-7: IC_{50} = 28.1 ± 0.42 µM NS2B/NS3: IC_{50} = 10 ± 2 µM DENV RNA: IC_{50} = 10 ± 2 µM 1 mg/kg increases the survival rate by 80%	Huh-7 cell: CC_{50} = >30 µM	Viral replication	Triggers Nrf2-HO-1 signaling (↑ interferon response / NS2B/NS3 protease inhibition)	[31]
**9**	Augusticornin B	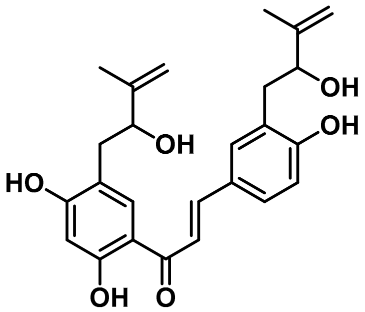	CHIKV	*In silico*	nsP2: E_{dock} = −115.9 kJ/mol	HsCASP3: E_{dock} = −111.2 kJ/mol	Not reported	nsP2 protease inhibitor	[32]
**10**	Isoliquiritigenin		Influenza A	*In vitro* *In vivo*	H1N1/ HBECs: EC_{50} = 24.7 µM 10 mg/kg decreases specific $CD8^+$ effector T cell infiltration in the lung of mice by 60%	HBECs: CC_{50} = 98.02 µM A549 cell: CC_{50} = 81.74 µM	Viral replication	PPARγ/Nrf2 pathway modulator, immune regulator	[35]

S/ N	Chalcones	Structure	Virus	Study Model	Inhibitory Concentration IC_{50}/EC_{50} Docking energy/score Selectivity/Therapeutic index SI/TI	Cytotoxic Concentration CC_{50}	Inhibition Stage	Target / Mode of action	Ref
		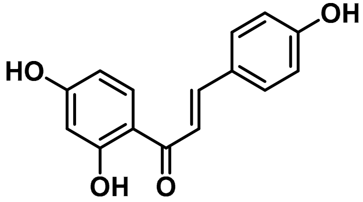	Influenza A	*In vitro*	H1N1 NA: $IC_{50} = 8.41 \pm 0.39$ µM<br>H9N2 NA: $IC_{50} = 9.69 \pm 0.37$ µM<br>H1N1(WT) NA: $IC_{50} = 3.48 \pm 0.19$ µM<br>H1N1(H274Y) NA: $IC_{50} = 3.42 \pm 0.12$ µM	Not reported	Not reported	Noncompetitive neuraminidase inhibitor	[36]
			HCV	*In vitro*	HCV/Huh7.5 cell: $IC_{50} = 3.7$ µg/mL /SI: 3.0	Huh7.5 cell: $CC_{50} = 11.0$ µg/mL	Post-entry stage	Not reported	[37]
**11**	Bavachalcone	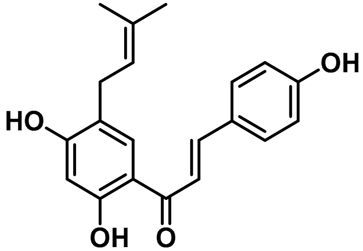	CHIKV	*In silico*	nsP2: $E_{dock} = -95.5$ kJ/mol	HsCASP3: $E_{dock} = -97.2$ kJ/mol	Not reported	nsP2 protease inhibitor	[32]
**12**	Isobavachalcone	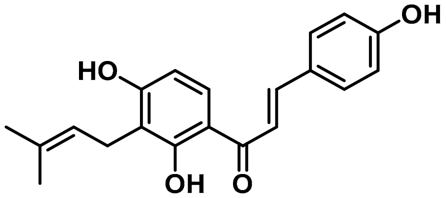	SARS-CoV-2	*In vitro*	$3CL^{pro}$ cell-free cleavage: $IC_{50} = 39.4 \pm 5.2$ µM /$Ki = 57.8 \pm 15.1$ µM (competitive)<br>$3CL^{pro}$ cell-based cleavage (Vero cell): $IC_{50} = 11.9 \pm 2.8$ µM /SI = 1.3<br>PL^{pro}: $IC_{50} = 13.0 \pm 0.9$ µM /$Ki = 7.9 \pm 1.4$ µM (mixed)	Mock cell: $CC_{50} = 15.3 \pm 1.6$ µM	Viral replication	3CL and PL protease inhibitor	[38]
			PRRSV	*In vitro*	HuN4 /Marc145 cell: $IC_{50} = 3.12$ µM /SI = 22.02	Marc145 cell: $CC_{50} = 63.09$ µM	Post-entry	Viral RNA replication inhibitor	[39]
**13**	Xanthohumol	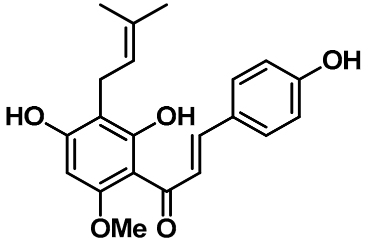	HCV	*In vitro*	Not reported	Not reported	Not reported	Reduces hepatic inflammation, steatosis and fibrosis through multiple targets	[81]
			SARS-CoV-2	*In vitro* *In silico*	SARS-CoV-2 /Caco-2 cell: $IC_{50} = 3.3$ µM /TI = 3.73<br>PL^{pro}: $IC_{50} = 162 \pm 46$<br>PDB ID: 6WX4 (PL^{pro}) /$D_{score} = -73.37$	Caco-2 cell: $CC_{50} = 12.3$ µM	Viral replication	PL protease partial inhibitor	[40]

S/N	Chalcones	Structure	Virus	Study Model	Inhibitory Concentration IC_{50}/EC_{50} Docking energy/score Selectivity/Therapeutic index SI/TI	Cytotoxic Concentration CC_{50}	Inhibition Stage	Target / Mode of action	Ref
**14**	5-Prenylbutein	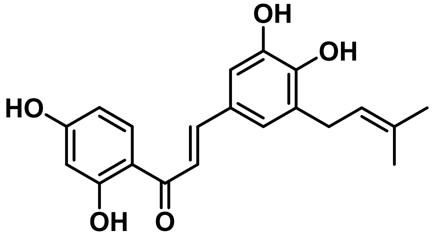	Influenza A	*In vitro*	H1N1 NA: IC_{50} = 25.87 ± 2.03 µM<br>H9N2 NA: IC_{50} = 35.50 ± 1.43 µM	Not reported	Not reported	Noncompetitive neuraminidase inhibitor	[36]
**15**	Butein	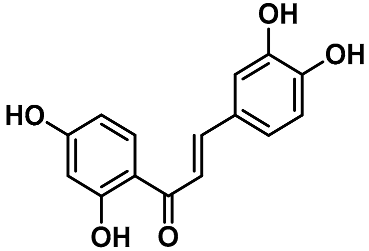	SARS-CoV-2	*In silico*	PDB ID: 6VW1 (*h*ACE2) /D_{score} = −7.38 kcal/mol	Not reported	Viral entry	*h*ACE2 binding	[97]
**16**	Broussochalcone A	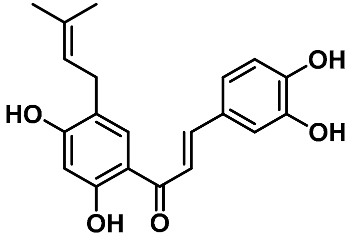	SARS-CoV-1 & -2	*In silico*	PDB ID: 6LU7 ($3CL^{pro}$ SARS-CoV-2)<br>Binding Affinity: −8 ± 0.10 kcal/mol<br>PDB ID: 5N5O ($3CL^{pro}$ SARS-CoV-1)<br>Binding Affinity: −7.7 ± 0.06 kcal/mol	Not reported	Viral replication	3CL protease inhibitor	[89]
**17**	Licoagrochalcone A	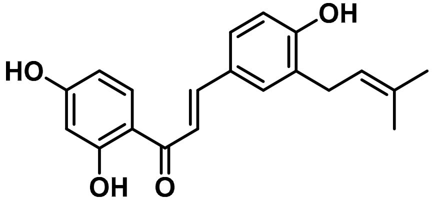	Influenza A	*In vitro*	H1N1 NA: IC_{50} = 51.59 ± 2.77 µM<br>H9N2 NA: IC_{50} = 56.92 ± 2.15 µM	Not reported	Not reported	Noncompetitive neuraminidase inhibitor	[36]
**18**	Kanzonol C	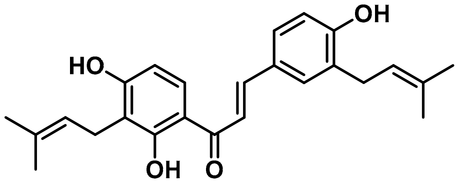	Influenza A	*In vitro*	H1N1 NA: IC_{50} = 75.38 ± 2.47 µM<br>H9N2 NA: IC_{50} = 52.96 ± 1.33 µM	Not reported	Not reported	Noncompetitive neuraminidase inhibitor	[36]

S/ N	Chalcones	Structure	Virus	Study Model	Inhibitory Concentration IC_{50}/EC_{50}<br>Docking energy/score<br>Selectivity/Therapeutic index SI/TI	Cytotoxic Concentration CC_{50}	Inhibition Stage	Target / Mode of action	Ref
**19**	Echinatin	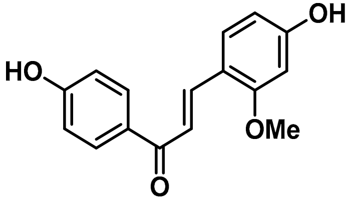	Influenza A	*In vitro*	H1N1 NA: IC_{50} = 5.80 ± 0.30 μM<br>H9N2 NA: IC_{50} = 5.70 ± 0.55 μM<br>H1N1(WT) NA: IC_{50} = 2.49 ± 0.14 μM<br>H1N1(H274Y) NA: IC_{50} = 2.19 ± 0.06 μM	Not reported	Not reported	Noncompetitive neuraminidase inhibitor	[36]
			SARS-CoV-2	*In vitro*	SARS-CoV-2 /Vero E6 cell: EC_{50} = 7.862 μM /SI = 15.27	Vero E6: CC_{50} = 120.1 μM	Post-entry	Nucleocapsid Protein inhibitor	[41]
**20**	Licochalcone A	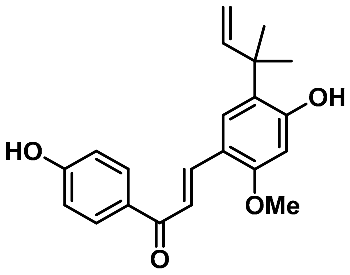	CHIKV	*In silico*	nsP2: E_{dock} = −106.4 kJ/mol	HsCASP3: E_{dock} = −95.7 kJ/mol	Not reported	nsP2 protease inhibitor	[32]
			EV-D68	*In vitro*	EV-D68/RD cell: EC_{50} = 3.62 μM	RD cell: CC_{50} = 35.20 μM	Early stage of viral replication	Viral IRES-driven translation suppressor	[42]
			EV-A71	*In vitro*<br>*In vivo*	EV-A71/RD cell: EC_{50} = 9.30 μM<br>EV-A71/Vero cell: EC_{50} = 5.73 μM<br>20 mg/kg improves the survival rates of ICR mice by 90%	RD cell: CC_{50} = 32.6 μM<br>Vero cell: CC_{50} = 30.2 μM	Early stage of viral replication	Not reported	[43]
			Influenza A	*In vitro*	H1N1 NA: IC_{50} = 19.09 ± 1.10 μM<br>H9N2 NA: IC_{50} = 17.98 ± 0.97 μM<br>H1N1(WT) NA: IC_{50} = 5.42 ± 0.40 μM<br>H1N1(H274Y) NA: IC_{50} = 4.20 ± 0.57 μM	Not reported	Not reported	Noncompetitive neuraminidase inhibitor	[36]
			HCV	*In vitro*	HCV/Huh7.5 cell: IC_{50} = 2.5 μg/mL /SI: 8.0	Huh7.5 cell: CC_{50} = 20.0 μg/mL	Post-entry stage	Not reported	[37]
			HSV	*In vitro*	HSV2/Vero cell: IC_{50} = 1.73 ± 0.04 μM /SI = 13.23	Vero cell: CC_{50} = 22.89 ± 0.35 μM	Viral replication	Not reported	[44]

S/ N	Chalcones	Structure	Virus	Study Model	Inhibitory Concentration IC_{50}/EC_{50} Docking energy/score Selectivity/Therapeutic index SI/TI	Cytotoxic Concentration CC_{50}	Inhibition Stage	Target / Mode of action	Ref
**21**	Licochalcone B	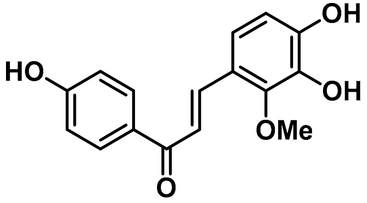	HSV	*In vitro* *In vivo*	HSV2/Vero cell: IC_{50} = 3.80 ± 0.23 μM /SI = 53.42 HSV2 ACV-R/Vero cell: IC_{50} = 3.18 ± 0.23 μM /SI = 63.8 Plaque assay with 80 mg/kg/d (LCB) ≈ 50 mg/kg/d (Positive control - ACV)	Vero cell: CC_{50} = 203.67 ± 3.92 μM	Post-infection /Viral replication	PI3K-Akt pathway inhibition	[44]
			SARS-CoV-2	*In vitro*	SARS-CoV-2 /Vero E6 cell: EC_{50} = 15.53 μM /SI = 6.86	Vero E6: CC_{50} = 106.5 μM	Post-entry	Nucleocapsid Protein inhibitor	[41]
**22**	Licochalcone D	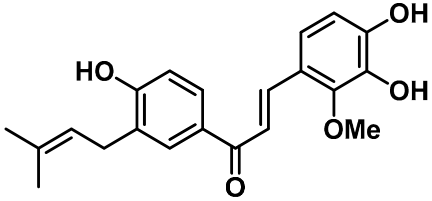	Influenza A	*In vitro*	H1N1 NA: IC_{50} = 28.62 ± 1.67 μM H9N2 NA: IC_{50} = 35.21 ± 3.10 μM	Not reported	Not reported	Noncompetitive neuraminidase inhibitor	[36]
**23**	Licochalcone G	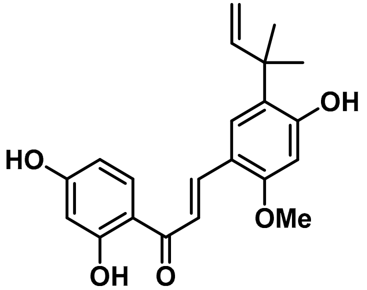	Influenza A	*In vitro*	H1N1 NA: IC_{50} = 37.68 ± 2.17 μM H9N2 NA: IC_{50} = 42.11 ± 2.12 μM	Not reported	Not reported	Noncompetitive neuraminidase inhibitor	[36]
**24**	4-Hydroxyderricin	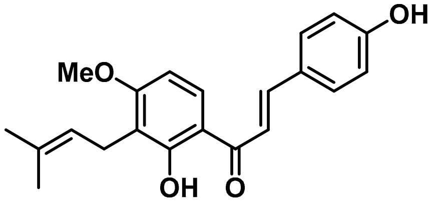	Influenza A	*In vitro*	H1N1 NA: IC_{50} = 42.1 ± 1.8 μM NA: *Ki* = 50.2 ± 5.7 μM (noncompetitive)	Not reported	Not reported	Noncompetitive neuraminidase inhibitor	[45]
			ZIKV	*In vitro* *In silico*	ZIKV/Vero cell: EC_{50} = 6.6 μM /SI: 17 NS2B/$NS3^{pro}$: IC_{50} = 47 ± 10 μM $NS3^{pro}/D_{score}$ = −8.0 kcal/mol $NS5^{RdRp}/D_{score}$ = −3.1 kcal/mol	Vero cell: CC_{50} = 103 ± 14 μM	Viral replication	Act partially as ZIKV NS3pro noncompetitive inhibitor	[33]

S/N	Chalcones	Structure	Virus	Study Model	Inhibitory Concentration IC_{50}/EC_{50}<br>Docking energy/score<br>Selectivity/Therapeutic index SI/TI	Cytotoxic Concentration CC_{50}	Inhibition Stage	Target / Mode of action	Ref
			SARS-CoV-2	*In vitro*	$3CL^{pro}$ cell-free cleavage: $IC_{50} = 81.4 \pm 8.5$ µM /$Ki = 85.9 \pm 11.6$ µM (competitive)<br>$3CL^{pro}$ cell-based cleavage (Vero cell): $IC_{50} = 50.8 \pm 3.0$ µM /SI = 0.4<br>PL^{pro}: $IC_{50} = 26.0 \pm 1.5$ µM /$Ki = 15.2 \pm 0.7$ µM (noncompetitive)	Mock cell: $CC_{50} = 23.0 \pm 2.4$ µM	Viral replication	3CL and PL protease inhibitor	[38]
**25**	Xanthoangelol	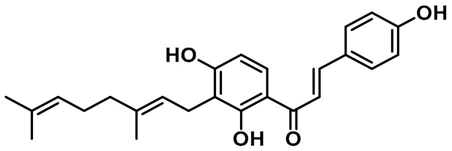	ZIKV	*In vitro*<br>*In silico*	ZIKV/Vero cell: EC_{50} = > tested<br>NS2B/NS3^{pro}: $IC_{50} = 50 \pm 5$ µM<br>NS5^{RdRp}: $IC_{50} = 6.9 \pm 0.9$ µM NS3/$D_{score} = -7.6$ kcal/mol<br>NS5^{RdRp}/$D_{score} = -3.8$ kcal/mol	Vero cell: CC_{50} = > tested	Not reported	Noncompetitive NS3pro inhibitor and NS5 RdRp inhibitor	[33]
			SARS-CoV-2	*In vitro*	$3CL^{pro}$ cell-free cleavage: $IC_{50} = 38.4 \pm 3.9$ µM /$Ki = 89.9 \pm 24.5$ µM (competitive)<br>$3CL^{pro}$ cell-based cleavage (Vero cell): $IC_{50} = 5.8 \pm 0.6$ µM /SI = 3.5<br>PL^{pro}: $IC_{50} = 11.7 \pm 3.2$ µM /$Ki = 10.7 \pm 0.7$ µM (noncompetitive)	Mock cell: $CC_{50} = 20.4 \pm 3.8$ µM	Viral replication	3CL and PL protease inhibitor	[38]
**26**	Xanthoangelol B	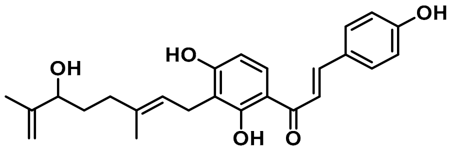	Influenza A	*In vitro*	H1N1 NA: $IC_{50} = 22.5 \pm 2.2$ µM<br>NA: $Ki = 20.7 \pm 1.1$ µM (noncompetitive)	Not reported	Not reported	Noncompetitive neuraminidase inhibitor	[45]
			SARS-CoV-2	*In vitro*	$3CL^{pro}$ cell-free cleavage: $IC_{50} = 22.2 \pm 6.5$ µM /$Ki = 43.8 \pm 8.2$ µM (competitive)<br>$3CL^{pro}$ cell-based cleavage (Vero cell): $IC_{50} = 8.6 \pm 2.6$ µM /SI = 4.2<br>PL^{pro}: $IC_{50} = 11.7 \pm 0.3$ µM /$Ki = 7.3 \pm 0.3$ µM (noncompetitive)	Mock cell: $CC_{50} = 36.3 \pm 0.6$ µM	Viral replication	3CL and PL protease inhibitor	[38]
**27**	Xanthoangelol D	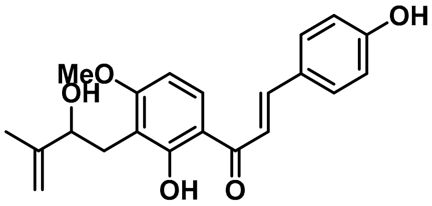	Influenza A	*In vitro*	H1N1 NA: $IC_{50} = 12.3 \pm 1.1$ µM<br>NA: $Ki = 14.0 \pm 1.5$ µM (noncompetitive)	Not reported	Not reported	Noncompetitive neuraminidase inhibitor	[45]

S/N	Chalcones	Structure	Virus	Study Model	Inhibitory Concentration IC_{50}/EC_{50} Docking energy/score Selectivity/Therapeutic index SI/TI	Cytotoxic Concentration CC_{50}	Inhibition Stage	Target / Mode of action	Ref
			SARS-CoV-2	*In vitro*	$3CL^{pro}$ cell-free cleavage: IC_{50} = 26.6 ± 5.2 µM /*Ki* = 34.8 ± 7.9 µM (competitive)<br>$3CL^{pro}$ cell-based cleavage (Vero cell): IC_{50} = 9.3 ± 1.2 µM /SI = 6.7<br>PL^{pro}: IC_{50} = 19.3 ± 1.8 µM /*Ki* = 10.7 ± 1.4 µM (noncompetitive)	Mock cell: CC_{50} = 62.0 ± 1.3 µM	Viral replication	3CL and PL protease inhibitor	[38]
**28**	Xanthoangelol E	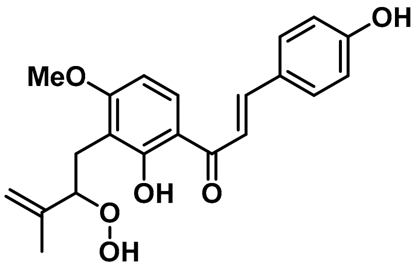	ZIKV	*In vitro*<br>*In silico*	ZIKV/Vero cell: EC_{50} = 22 µM /SI: 5<br>NS2B/$NS3^{pro}$: IC_{50} = 18 ± 5 µM<br>$NS3^{pro}$/D_{score} = −8.0 kcal/mol<br>$NS5^{RdRp}$/D_{score} = −4.4 kcal/mol	Vero cell: CC_{50} = 111 ± 10 µM	Viral replication	Noncompetitive ZIKV NS3pro inhibitor	[33]
			SARS-CoV-2	*In vitro*	$3CL^{pro}$ cell-free cleavage: IC_{50} = 11.4 ± 1.4 µM /*Ki* = 16.1 ± 2.5 µM (competitive)<br>$3CL^{pro}$ cell-based cleavage (Vero cell): IC_{50} = 7.1 ± 0.8 µM /SI = 9.2<br>PL^{pro}: IC_{50} = 1.2 ± 0.4 µM /*Ki* = 1.2 ± 0.06 µM (noncompetitive)	Mock cell: CC_{50} = 65.6 ± 1.7 µM	Viral replication	3CL and PL protease inhibitor	[38]
**29**	Xanthoangelol F	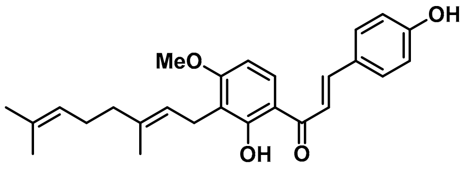	Influenza A	*In vitro*	H1N1 NA: IC_{50} = 85.9 ± 5.0 µM<br>NA: *Ki* = 122.7 ± 8.0 µM (noncompetitive)	Not reported	Not reported	Noncompetitive neuraminidase inhibitor	[45]
			SARS-CoV-2	*In vitro*	$3CL^{pro}$ cell-free cleavage: IC_{50} = 34.1 ± 4.8 µM /*Ki* = 29.2 ± 11.7 µM (competitive)<br>$3CL^{pro}$ cell-based cleavage (Vero cell): IC_{50} = 32.6 ± 2.2 µM /SI = 0.6<br>PL^{pro}: IC_{50} = 5.6 ± 0.5 µM /*Ki* = 5.4 ± 1.7 µM (noncompetitive)	Mock cell: CC_{50} = 28.8 ± 6.3 µM	Viral replication	3CL and PL protease inhibitor	[38]
**30**	Xanthoangelol G		Influenza A	*In vitro*	H1N1 NA: IC_{50} = 24.2 ± 0.7 µM<br>NA: *Ki* = 20.0 ± 2.2 µM (noncompetitive)	Not reported	Not reported	Noncompetitive neuraminidase inhibitor	[45]

S/N	Chalcones	Structure	Virus	Study Model	Inhibitory Concentration IC_{50}/EC_{50} Docking energy/score Selectivity/Therapeutic index SI/TI	Cytotoxic Concentration CC_{50}	Inhibition Stage	Target / Mode of action	Ref
		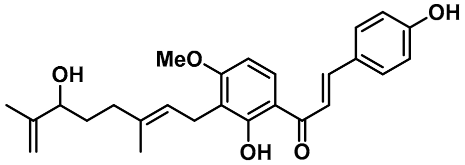	SARS-CoV-2	*In vitro*	$3CL^{pro}$ cell-free cleavage: IC_{50} = 129.8 ±10.3 µM /*Ki* = 267.3 ± 50.1 µM (competitive)<br>PL^{pro}: IC_{50} = 46.4 ± 7.8 µM /*Ki* = 32.5 ± 2.4 µM (noncompetitive)	Not reported	Viral replication	3CL and PL protease inhibitor	[38]
**31**	Xanthokeistal A	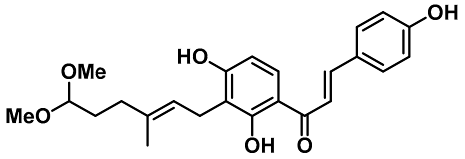	Influenza A	*In vitro*	H1N1 NA: IC_{50} = 30.9 ± 2.1 µM<br>NA: *Ki* = 33.5 ± 3.0 µM (noncompetitive)	Not reported	Not reported	Noncompetitive neuraminidase inhibitor	[45]
			SARS-CoV-2	*In vitro*	$3CL^{pro}$ cell-free cleavage: IC_{50} = 44.1 ± 1.3 µM /*Ki* = 76.1 ± 12.0 µM (competitive)<br>$3CL^{pro}$ cell-based cleavage (Vero cell): IC_{50} = 9.8 ± 2.3 µM /SI = 6.4<br>PL^{pro}: IC_{50} = 21.1 ± 5.6 µM /*Ki* = 12.9 ± 0.5 µM (noncompetitive)	Mock cell: CC_{50} = 62.6 ± 6.3 µM	Viral replication	3CL and PL protease inhibitor	[38]
**32**	2', 4'-dihydroxy-4-methoxy chalcone	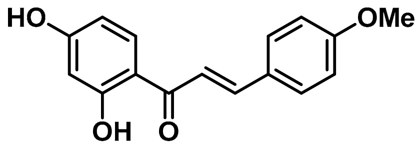	Influenza A	*In vitro*	H1N1 NA: IC_{50} = 2.23 µM /SI: >224.2	MDCK cell: >500 µM	Not reported	Noncompetitive neuraminidase inhibitor	[53]
**33**	2', 4'-dihydroxy-3-methoxy chalcone	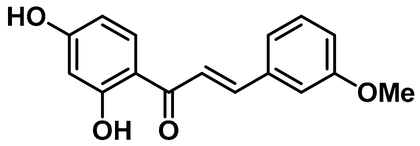	Influenza A	*In vitro*	H1N1 NA: IC_{50} = 8.71 µM /SI: >57.4	MDCK cell: >500 µM	Not reported	Noncompetitive neuraminidase inhibitor	[53]
**34**	2', 4'-dihydroxy-3-chloro chalcone	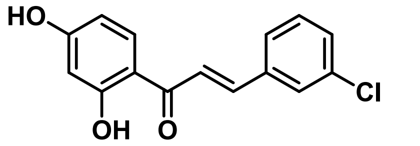	Influenza A	*In vitro*	H1N1 NA: IC_{50} = 3.58 µM /SI: >69.8	MDCK cell: >250 µM	Not reported	Noncompetitive neuraminidase inhibitor	[53]
**35**	2', 4'-dihydroxy-4-nitro chalcone	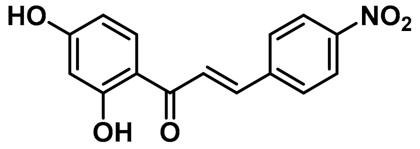	Influenza A	*In vitro*	H1N1 NA: IC_{50} = 6.64 µM /SI: >18.8	MDCK cell: >125 µM	Not reported	Noncompetitive neuraminidase inhibitor	[53]

S/N	Chalcones	Structure	Virus	Study Model	Inhibitory Concentration IC_{50}/EC_{50} Docking energy/score Selectivity/Therapeutic index SI/TI	Cytotoxic Concentration CC_{50}	Inhibition Stage	Target / Mode of action	Ref
**36**	1a (R = 2-Cl)	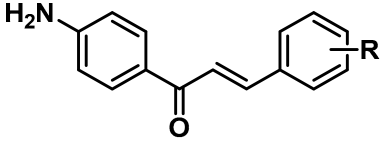	Influenza A	*In vitro*	H1N1/MDCK cell: EC_{50} = 2.36 nM /SI: 72,288<br>H1N1 NA: IC_{50} = 6.64 µM	MDCK cell: CC_{50} = 170.6 µM	Viral replication	Noncompetitive neuraminidase inhibitor	[54]
**37**	1b (R = 3-Cl)		Influenza A	*In vitro*	H1N1/MDCK cell: EC_{50} = 2.36 nM /SI: 63,729<br>H1N1 NA: IC_{50} = 5.56 µM	MDCK cell: CC_{50} = 150.4 µM	Viral replication	Noncompetitive neuraminidase inhibitor	[54]
**38**	1e (R = 3-OMe)		Influenza A	*In vitro*	H1N1/MDCK cell: EC_{50} = 1.71 nM /SI: 105,497<br>H1N1 NA: IC_{50} = 4.10 µM	MDCK cell: CC_{50} = 180.4 µM	Viral replication	Noncompetitive neuraminidase inhibitor	[54]
**39**	1f (R = 4-OMe)		Influenza A	*In vitro*	H1N1/MDCK cell: EC_{50} = 2.76 nM /SI: 72,428<br>H1N1 NA: IC_{50} = 3.58 µM	MDCK cell: CC_{50} = 199.9 µM	Viral replication	Noncompetitive neuraminidase inhibitor	[54]
**40**	1h (R = 3-NO_2)		Influenza A	*In vitro*	H1N1/MDCK cell: EC_{50} = 5.26 nM /SI: 24,316<br>H1N1 NA: IC_{50} = 8.26 µM	MDCK cell: CC_{50} = 127.9 µM	Viral replication	Noncompetitive neuraminidase inhibitor	[54]
**41**	1i (R = 4-NO_2)		Influenza A	*In vitro*	H1N1/MDCK cell: EC_{50} = 4.62 nM /SI: 24,372<br>H1N1 NA: IC_{50} = 7.86 µM	MDCK cell: CC_{50} = 112.6 µM	Viral replication	Noncompetitive neuraminidase inhibitor	[54]
**42**	2c (R = 4-Cl)	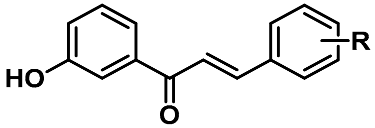	Influenza A	*In vitro*	H1N1/MDCK cell: EC_{50} = 15.64 nM /SI: 7,417<br>H1N1 NA: IC_{50} = 12.36 µM	MDCK cell: CC_{50} = 116.0 µM	Viral replication	Noncompetitive neuraminidase inhibitor	[54]
**43**	2f (R = 4-OMe)		Influenza A	*In vitro*	H1N1/MDCK cell: EC_{50} = 8.62 nM /SI: 33,921<br>H1N1 NA: IC_{50} = 5.25 µM	MDCK cell: CC_{50} = 292.4 µM	Viral replication	Noncompetitive neuraminidase inhibitor	[54]

S/N	Chalcones	Structure	Virus	Study Model	Inhibitory Concentration IC_{50}/EC_{50} Docking energy/score Selectivity/Therapeutic index SI/TI	Cytotoxic Concentration CC_{50}	Inhibition Stage	Target / Mode of action	Ref
**44**	2i (R = 4-NO_2)		Influenza A	*In vitro*	H1N1/MDCK cell: EC_{50} = 12.65 nM /SI: 13,423<br>H1N1 NA: IC_{50} = 8.65 μM	MDCK cell: CC_{50} = 169.8 μM	Viral replication	Noncompetitive neuraminidase inhibitor	[54]
**45**	1c-H	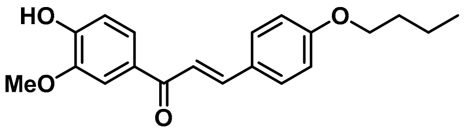	Influenza A	*In vitro*<br>*In silico*	H1N1 NA: IC_{50} = 28.11 ± 1.88 μM<br>H5N1 NA: IC_{50} = 27.63 ± 2.41 μM<br>H1N1 NA: ΔG_{bind} = −6.72 kcal/mol, *Ki* = 11.82 μM<br>H5N1 NA: ΔG_{bind} = −6.53 kcal/mol, *Ki* = 18.30 μM	Vero cell: CC_{50} = 968.16 ± 5.05 μM	Not reported	Noncompetitive neuraminidase inhibitor	[55]
**46**	2b-H	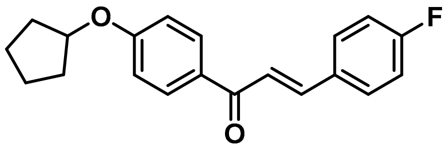	Influenza A	*In vitro*<br>*In silico*	H1N1 NA: IC_{50} = 87.54 ± 5.74 μM<br>H5N1 NA: IC_{50} = 73.17 ± 5.87 μM<br>H1N1 NA: ΔG_{bind} = −6.18 kcal/mol, *Ki* = 29.34 μM<br>H5N1 NA: ΔG_{bind} = −7.32 kcal/mol, *Ki* = 4.33 μM	Vero cell: CC_{50} = 757.18 ± 3.91 μM	Not reported	Noncompetitive neuraminidase inhibitor	[55]
**47**	A9	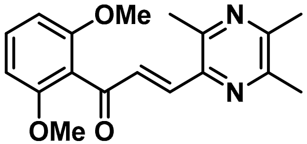	Influenza A	*In vitro*	H1N1 pdm09/ CEF cell: EC_{50} = 7.34 μM /SI: 30.9	CEF cell: CC_{50} = 41.46 μM	Viral replication	Not reported	[56]
**48**	6’-O-rhamnosyl-(1’’’ → 6’’)-glucopyranosyl asebogenin (Thalassodendrone)	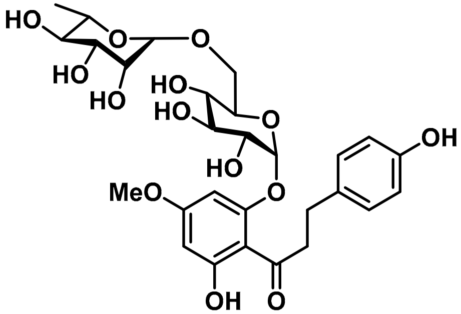	Influenza A	*In vitro*	H1N1(WSN/33)/MDCK cell: IC_{50} = 2.00 μg/mL /SI: 1.68	MDCK cell: 3.36 μg/mL	Viral replication	Not reported	[46]

S/ N	Chalcones	Structure	Virus	Study Model	Inhibitory Concentration IC_{50}/EC_{50} Docking energy/score Selectivity/Therapeutic index SI/TI	Cytotoxic Concentration CC_{50}	Inhibition Stage	Target / Mode of action	Ref
**49**	Asebotin	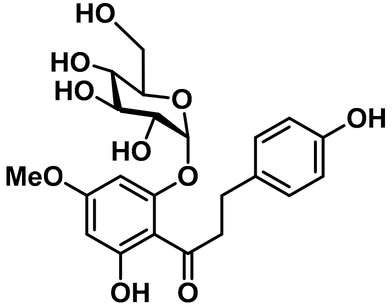	Influenza A	*In vitro*	H1N1(WSN/33)/MDCK cell: IC_{50} = 1.96 μg/mL/SI: 1.60	MDCK cell: 3.14 μg/mL	Viral replication	Not reported	[46]
**50**	3h	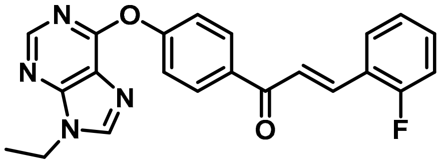	TMV CMV	*In vitro* *In vivo*	Curative, protective, and inactivation activity against TMV at 500 μg/mL = 41%; 45%; 48%. Curative EC_{50} = 407.9 μg/mL. TMV-CP K_d = 6.7 mM Curative and protective activity against CMV at 500 μg/mL = 49%; 39%.	Not reported	Not reported	TMV-CP interaction	[60]
**51**	3o	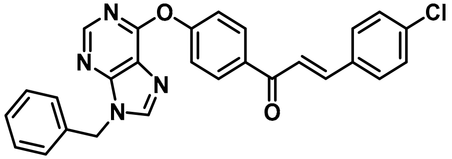	TMV CMV	*In vitro* *In vivo*	Curative, protective, and inactivation activity against TMV at 500 μg/mL = 42%; 46%; 89%. Curative EC_{50} = 301.1 μg/mL. TMV-CP K_d = 5.1 μM Curative and protective activity against CMV in 500 μg/mL = 58%; 52%.	Not reported	Not reported	TMV-CP interaction	[60]
**52**	3s	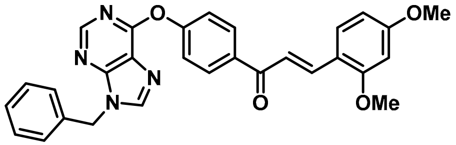	TMV CMV	*In vitro* *In vivo*	Curative, protective, and inactivation activity against TMV at 500 μg/mL = 42%; 40%; 75%. Curative EC_{50} = 315.7 μg/mL. TMV-CP K_d = 155 μM Curative and protective activity against CMV at 500 μg/mL = 53%; 46%.	Not reported	Not reported	TMV-CP interaction	[60]
**53**	3w	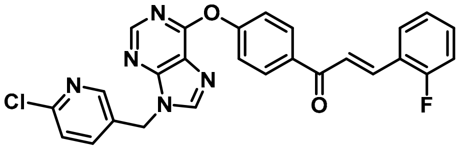	TMV CMV	*In vitro* *In vivo*	Curative, protective, and inactivation activity against TMV at 500 μg/mL = 46%; 56%; 64%. Curative EC_{50} = 282.3 μg/mL Curative and protective activity against CMV at 500 μg/mL = 53%; 45%.	Not reported	Not reported	Potentially TMV-CP interaction	[60]
**54**	3x	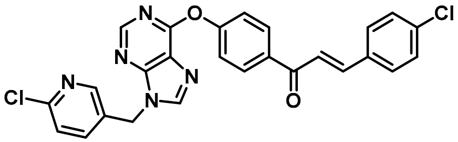	TMV CMV	*In vitro* *In vivo*	Curative, protective, and inactivation activity against TMV at 500 μg/mL = 49%; 42%; 59%. Curative EC_{50} = 230.5 μg/mL Curative and protective activity against CMV at 500 μg/mL = 59%; 42%.	Not reported	Not reported	Potentially TMV-CP interaction	[60]

S/N	Chalcones	Structure	Virus	Study Model	Inhibitory Concentration IC_{50}/EC_{50} Docking energy/score Selectivity/Therapeutic index SI/TI	Cytotoxic Concentration CC_{50}	Inhibition Stage	Target / Mode of action	Ref
**55**	3n’	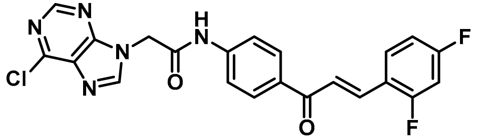	TMV	*In vitro* *In vivo*	EC_{50} values for curative, protective, and inactivation activity against TMV (μg/mL) = 452; 416; 241. TMV-CP K_d = 79.8 μM	Not reported	Not reported	TMV-CP interaction	[70]
**56**	3p’	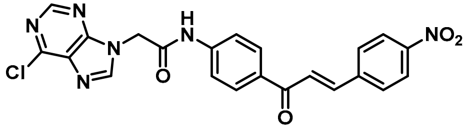	TMV	*In vitro* *In vivo*	EC_{50} values for curative, protective, and inactivation activity against TMV (μg/mL) = 439; 419; 262.	Not reported	Not reported	Potentially TMV-CP interaction	[70]
**57**	d2	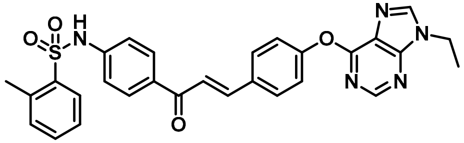	TMV CMV	*In vitro* *In vivo*	Curative, protective, and inactivation activity against TMV at 500 μg/mL = 56%; 57%; 89%. Inactivation EC_{50} = 51.7 μg/mL; TMV-CP K_d = 12.16 μM Curative, protective, and inactivation activity against CMV at 500 μg/mL = 56%; 57%; 66%	Not reported	Not reported	TMV-CP interaction	[61]
**58**	d7	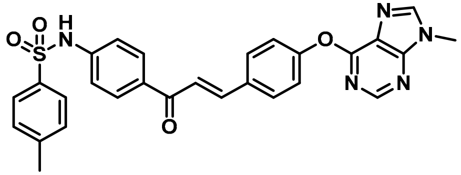	TMV CMV	*In vitro* *In vivo*	Curative, protective, and inactivation activity against TMV at 500 μg/mL = 55%; 64%; 89%. Inactivation EC_{50} = 53.5 μg/mL; TMV-CP K_d = 20.63 μM Curative, protective, and inactivation activity against CMV at 500 μg/mL = 53%; 56%; 63%	Not reported	Not reported	TMV-CP interaction	[61]
**59**	7g	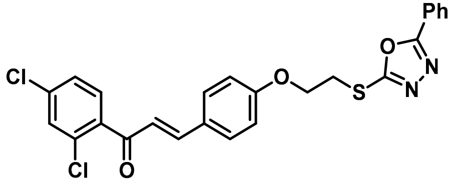	TMV	*In vitro* *In vivo*	TMV inhibition ratio at 500 μg/mL = 95% EC_{50} = 33.66 μg/mL TMV-CP K_d = 5.93 μM	Not reported	Not reported	TMV-CP interaction	[59]
**60**	7l	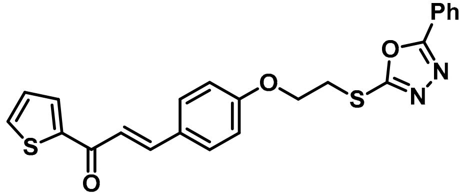	TMV	*In vitro* *In vivo*	TMV inhibition ratio at 500 μg/mL = 93% EC_{50} = 33.97 μg/mL TMV-CP K_d = 6.15 μM	Not reported	Not reported	TMV-CP interaction	[59]

S/N	Chalcones	Structure	Virus	Study Model	Inhibitory Concentration IC_{50}/EC_{50} Docking energy/score Selectivity/Therapeutic index SI/TI	Cytotoxic Concentration CC_{50}	Inhibition Stage	Target / Mode of action	Ref
**61**	8h	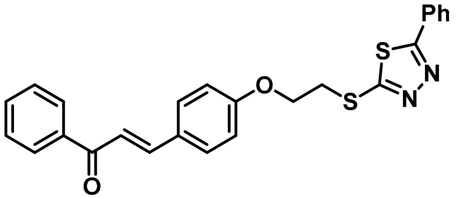	TMV	*In vitro* *In vivo*	TMV inhibition ratio at 500 µg/mL = 93% EC_{50} = 33.87 µg/mL TMV-CP K_d = 6.02 µM	Not reported	Not reported	TMV-CP interaction	[59]
**62**	8l	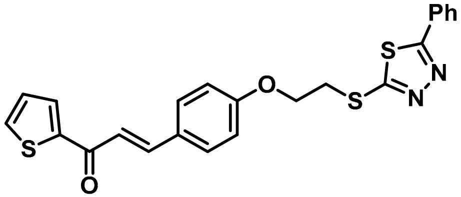	TMV	*In vitro* *In vivo*	TMV inhibition ratio at 500 µg/mL = 94% EC_{50} = 30.57 µg/mL TMV-CP K_d = 5.04 µM	Not reported	Not reported	TMV-CP interaction	[59]
**63**	5l	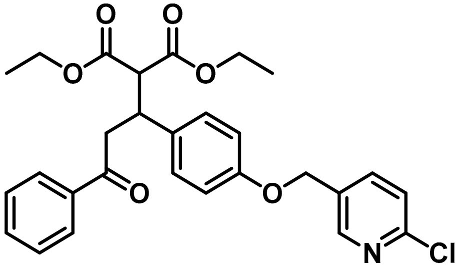	CMV	*In vitro* *In vivo*	CMV inhibition ratio at 500 µg/mL = 88% EC_{50} = 186.17 µg/mL	Not reported	Not reported	Not reported	[69]
**64**	5n	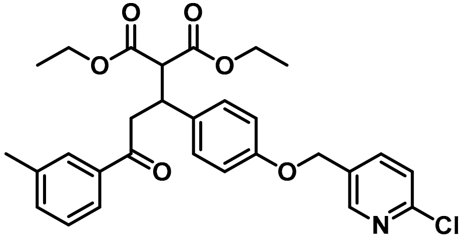	CMV	*In vitro* *In vivo*	CMV inhibition ratio at 500 µg/mL = 89% EC_{50} = 211.47 µg/mL	Not reported	Not reported	Not reported	[69]
**65**	H9	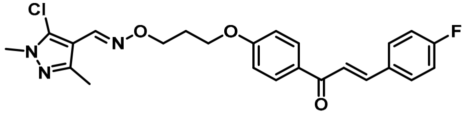	TMV	*In vitro* *In vivo*	Curative EC_{50} = 166.9 µg/mL Protective EC_{50} = 126.5 µg/mL TMV-CP K_d = 0.0096 ± 0.0045 µM	Not reported	Not reported	TMV-CP interaction	[62]

S/N	Chalcones	Structure	Virus	Study Model	Inhibitory Concentration IC_{50}/EC_{50}<br>Docking energy/score<br>Selectivity/Therapeutic index SI/TI	Cytotoxic Concentration CC_{50}	Inhibition Stage	Target / Mode of action	Ref
**66**	N22	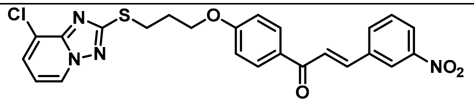	TMV	*In vitro*<br>*In vivo*	Curative EC_{50} = 77.64 µg/mL<br>Protective EC_{50} = 81.55 µg/mL<br>Inactivation effect = 88%<br>TMV-CP K_d = 0.0076 ± 0.0007 µM	Not reported	Not reported	TMV-CP interaction	[63]
**67**	S14	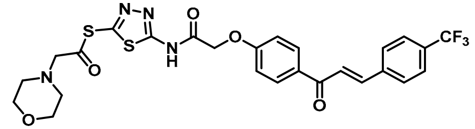	TMV	*In vitro*<br>*In vivo*	Curative EC_{50} = 91.8 µg/mL<br>Protective EC_{50} = 130.6 µg/mL<br>TMV-CP K_d = 0.0126 ± 0.0058 µM	Not reported	Not reported	TMV-CP interaction	[64]
**68**	S7	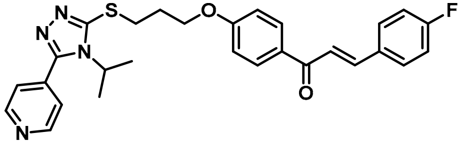	TMV	*In vitro*<br>*In vivo*	Curative EC_{50} = 89.7 µg/mL<br>Protective EC_{50} = 95.4 µg/mL<br>TMV-CP K_d = 0.5340 ± 0.2233 µM	Not reported	Not reported	TMV-CP interaction	[71]
**69**	T19	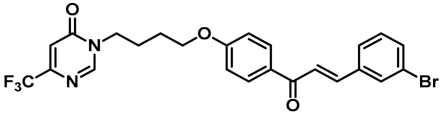	TMV	*In vitro*<br>*In vivo*	Curative EC_{50} = 234.9 µg/mL<br>Protective EC_{50} = 199.5 µg/mL<br>TMV-CP K_d = 0.0031 ± 0.0009 µM	Not reported	Not reported	TMV-CP interaction	[65]
**70**	D11	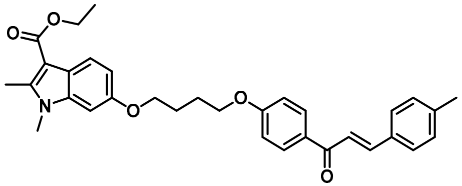	TMV	*In vitro*<br>*In vivo*	Curative EC_{50} = 107.4 µg/mL<br>Protective EC_{50} = 190.8 µg/mL<br>TMV-CP K_d = 0.0030 ± 0.0014 µM	Not reported	Not reported	TMV-CP interaction	[66]
**71**	Z15	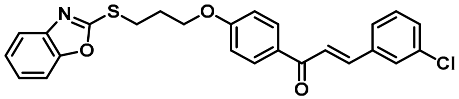	TMV	*In vitro*<br>*In vivo*<br>*In silico*	Curative EC_{50} = 101.97 µg/mL<br>Protective EC_{50} = 105.26 µg/mL	Not reported	Not reported	Potentially TMV-CP interaction	[67]
**72**	B3	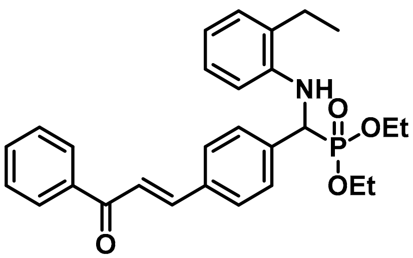	TMV	*In vitro*<br>*In vivo*	Curative EC_{50} = 356.7 µg/mL<br>TMV-CP K_a = 2.51 × 10^8 M^{-1}	Not reported	Not reported	TMV-CP interaction	[68]

S/N	Chalcones	Structure	Virus	Study Model	Inhibitory Concentration IC_{50}/EC_{50} Docking energy/score Selectivity/Therapeutic index SI/TI	Cytotoxic Concentration CC_{50}	Inhibition Stage	Target / Mode of action	Ref
**73**	1	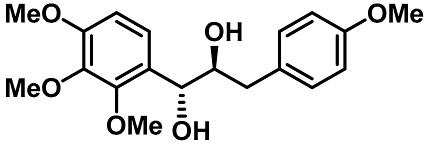	TMV	*In vitro*<br>*In vivo*	Inhibition ratio at 100 µg/mL = 80%	Not reported	Not reported	Not reported	[76]
**74**	2	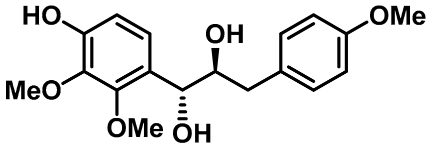	TMV	*In vitro*<br>*In vivo*	Inhibition ratio at 100 µg/mL = 62%	Not reported	Not reported	Not reported	[76]
**75**	4	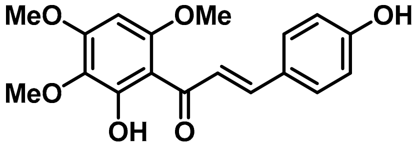	TMV	*In vitro*<br>*In vivo*	Anti-TMV: IC_{50} = 52.1 µM<br>Inhibition ratio at 20 µM = 25.8 ± 3.2	Not reported	Not reported	Not reported	[75]
**76**	L1	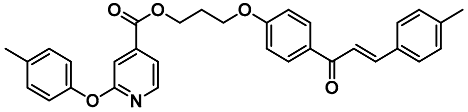	TMV	*In vitro*<br>*In vivo*<br>*In silico*	Curative EC_{50} = 140.5 µg/mL<br>Protective EC_{50} = 154.1 µg/mL	Not reported	Not reported	Potentially TMV-CP interaction	[72]
**77**	L4	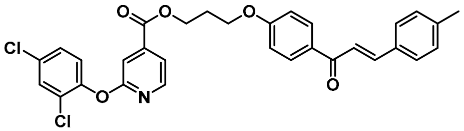	TMV	*In vitro*<br>*In vivo*<br>*In silico*	Curative EC_{50} = 90.7 µg/mL<br>Protective EC_{50} = 102.6 µg/mL<br>TMV-CP: *Kd* = 0.00149 ± 0.00071 µM	Not reported	Not reported	TMV-CP interaction	[72]
**78**	3-(2-hydroxy-4-methoxyphenyl)-1-(4-hydroxyphenyl)propan-1-one	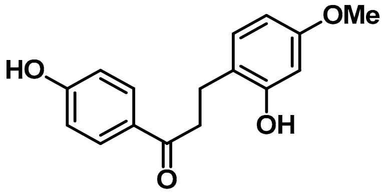	HBV	*In vitro*	HBsAg: IC_{50} = 20.56 µg/mL /TI: 16.86	HepG2.2.15 cell: CC_{50} = 346.67 µg/mL	Not reported	HBsAg and HBeAg inhibitor	[47]
**79**	(*E*)-3-(2-hydroxy-4-methoxyphenyl)-1-(4-hydroxyphenyl)prop-2-en-1-one	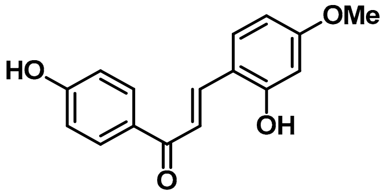	HBV	*In vitro*	HBsAg: IC_{50} = 6.36 µg/mL /TI: 38.08	HepG2.2.15 cell: CC_{50} = 242.11 µg/mL	Not reported	HBsAg and HBeAg inhibitor	[47]

S/N	Chalcones	Structure	Virus	Study Model	Inhibitory Concentration IC_{50}/EC_{50}<br>Docking energy/score<br>Selectivity/Therapeutic index SI/TI	Cytotoxic Concentration CC_{50}	Inhibition Stage	Target / Mode of action	Ref
**80**	15	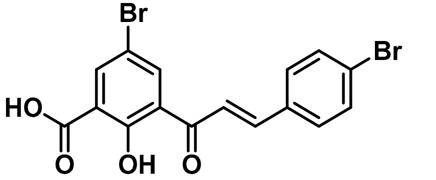	HIV-1	*In vitro*	HIV/PBM cell: EC_{50} = 8.7 µM /EC_{90} = 21.0 µM<br>HIV IN (3'-processing): IC_{50} = 11 ± 4<br>HIV IN (strand transfer): IC_{50} = 5 ± 2	PBM cell: IC_{50} = 24.4 µM<br>CEM cell: IC_{50} = 27.5 µM	Viral replication	Integrase inhibitor	[87]
**81**	25	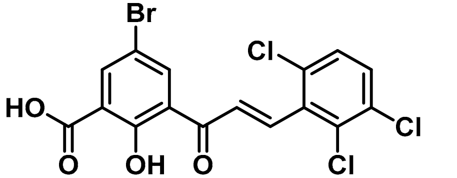	HIV-1	*In vitro*	HIV/PBM cell: EC_{50} = 7.3 µM /EC_{90} = 24.3 µM<br>HIV IN (strand transfer): IC_{50} = < 3.7	PBM cell: IC_{50} = 22.7 µM<br>CEM cell: IC_{50} = 29.0 µM	Viral replication	Integrase inhibitor	[87]
**82**	A1	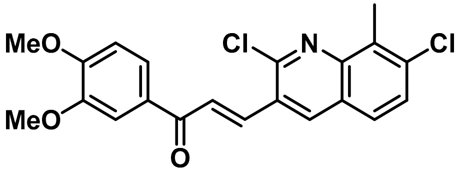	HIV-1	*In vitro*<br>*In silico*	HIV/PBM cell: EC_{50} = 5.78 µM /EC_{90} = 22.14 µM<br>HIV RT: IC_{50} = 0.15 µg/mL<br>HIV RT: E_{dock} = −8.38 kcal/mol<br>RT *ki* = 104.97 µM	PBM cell: IC_{50} = 40.01 µM<br>CEM cell: IC_{50} = 39.70 µM<br>Vero cell: IC_{50} = 27.31 µM	Not reported	Non-nucleoside reverse transcriptase inhibitor (NNRT)	[86]
**83**	A4 (R = Br)	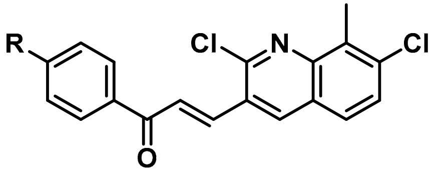	HIV-1	*In vitro*<br>*In silico*	HIV/PBM cell: EC_{50} = 1.43 µM /EC_{90} = 10.8 µM<br>HIV RT: IC_{50} = 0.10 µg/mL<br>HIV RT: E_{dock} = −10.05 kcal/mol<br>RT *ki* = 413.39 µM	PBM cell: IC_{50} = 32.09 µM<br>CEM cell: IC_{50} = 34.08 µM<br>Vero cell: IC_{50} = 28.07 µM	Not reported	Non-nucleoside reverse transcriptase inhibitor (NNRT)	[86]
**84**	A6 (R = Cl)		HIV-1	*In vitro*<br>*In silico*	HIV/PBM cell: EC_{50} = 1.58 µM /EC_{90} = 12.19 µM<br>HIV RT: IC_{50} = 0.11 µg/mL<br>HIV RT: E_{dock} = −10.13 kcal/mol<br>RT *ki* = 306.22 µM	PBM cell: IC_{50} = 22.99 µM<br>CEM cell: IC_{50} = 31.09 µM<br>Vero cell: IC_{50} = 24.73 µM	Not reported	Non-nucleoside reverse transcriptase inhibitor (NNRT)	[86]
**85**	A7 (R = OMe)		HIV-1	*In vitro*<br>*In silico*	HIV/PBM cell: EC_{50} = 7.78 µM /EC_{90} = 26.23 µM<br>HIV RT: IC_{50} = 0.25 µg/mL<br>HIV RT: E_{dock} = −9.50 kcal/mol<br>RT *ki* = 791.79 µM	PBM cell: IC_{50} = 50.05 µM<br>CEM cell: IC_{50} = 33.98 µM<br>Vero cell: IC_{50} = 31.90 µM	Not reported	Non-nucleoside reverse transcriptase inhibitor (NNRT)	[86]
**86**	A8	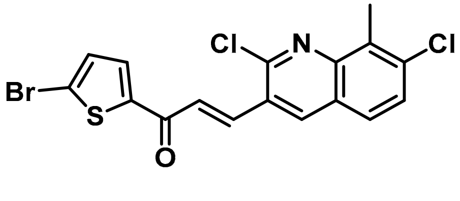	HIV-1	*In vitro*<br>*In silico*	HIV/PBM cell: EC_{50} = 1.98 µM /EC_{90} = 11.91 µM<br>HIV RT: IC_{50} = 0.14 µg/mL<br>HIV RT: E_{dock} = −9.56 kcal/mol<br>RT *ki* = 418.46 µM	PBM cell: IC_{50} = 27.01 µM<br>CEM cell: IC_{50} = 33.02 µM<br>Vero cell: IC_{50} = 29.01 µM	Not reported	Non-nucleoside reverse transcriptase inhibitor (NNRT)	[86]

S/N	Chalcones	Structure	Virus	Study Model	Inhibitory Concentration IC_{50}/EC_{50} Docking energy/score Selectivity/Therapeutic index SI/TI	Cytotoxic Concentration CC_{50}	Inhibition Stage	Target / Mode of action	Ref
**87**	A10	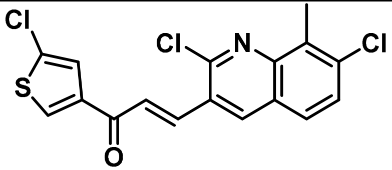	HIV-1	*In vitro* *In silico*	HIV/PBM cell: EC_{50} = 1.89 µM /EC_{90} = 14.09 µM HIV RT: IC_{50} = 0.13 µg/mL HIV RT: E_{dock} = −9.76 kcal/mol RT *ki* = 371.48 µM	PBM cell: IC_{50} = 24.80 µM CEM cell: IC_{50} = 28.90 µM Vero cell: IC_{50} = 26.50 µM	Not reported	Non-nucleoside reverse transcriptase inhibitor (NNRT)	[86]
**88**	A12	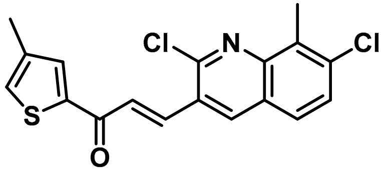	HIV-1	*In vitro* *In silico*	HIV/PBM cell: EC_{50} = 7.90 µM /EC_{90} = 37.09 µM HIV RT: IC_{50} = 0.19 µg/mL HIV RT: E_{dock} = −9.52 kcal/mol RT *ki* = 210.60 µM	PBM cell: IC_{50} = 55.99 µM CEM cell: IC_{50} = 62.04 µM Vero cell: IC_{50} = 46.78µM	Not reported	Non-nucleoside reverse transcriptase inhibitor (NNRT)	[86]
**89**	A13	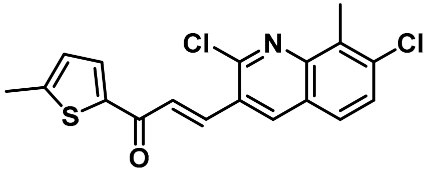	HIV-1	*In vitro* *In silico*	HIV/PBM cell: EC_{50} = 8.12 µM /EC_{90} = 40.09 µM HIV RT: IC_{50} = 0.18 µg/mL HIV RT: E_{dock} = −9.00 kcal/mol RT *ki* = 1.10 µM	PBM cell: IC_{50} = 51.89 µM CEM cell: IC_{50} = 63.03 µM Vero cell: IC_{50} = 39.90µM	Not reported	Non-nucleoside reverse transcriptase inhibitor (NNRT)	[86]
**90**	5h	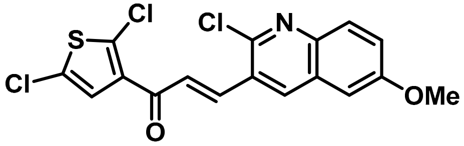	HIV-1	*In vitro*	HIV/PBM cell: EC_{50} = 1.1 µM /EC_{90} = 6.2 µM	PMB cell: IC_{50} = 2.7 µM CEM cell: IC_{50} = 6.5 µM Vero cell: IC_{50} = 4.6 µM	Not reported	Not reported	[88]
**91**	8l	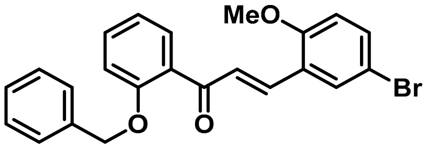	HIV-1	*In vitro*	HIV/PM1 cell inhibition ratio at 10 µM (%) = 99.9 ± 0.1 HIV/PM1 cell inhibition ratio at 5 µM (%) = 80.0 ± 13.9	Inhibition rate of PM1 cell viability at 10 µM (%) = 61.7 ± 5.8 Inhibition rate of PM1 cell viability at 5 µM (%) = 0.6 ± 7.2	Not reported	Not reported	[85]
**92**	8o	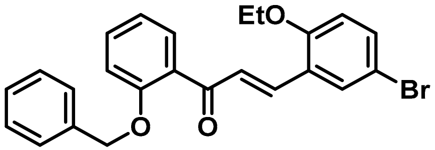	HIV-1	*In vitro*	HIV/PM1 cell inhibition ratio at 10 µM (%) = 92.3 ± 2.7 HIV/PM1 cell inhibition ratio at 5 µM (%) = 72.2 ± 9.2	Inhibition rate of PM1 cell viability at 10 µM (%) = None Inhibition rate of PM1 cell viability at 5 µM (%) = None	Not reported	Not reported	[85]

S/N	Chalcones	Structure	Virus	Study Model	Inhibitory Concentration IC_{50}/EC_{50} Docking energy/score Selectivity/Therapeutic index SI/TI	Cytotoxic Concentration CC_{50}	Inhibition Stage	Target / Mode of action	Ref
**93**	8p	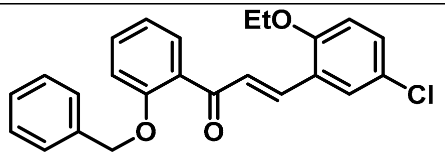	HIV-1	*In vitro*	HIV/PM1 cell inhibition ratio at 10 µM (%) = 95.8 ± 1.7<br>HIV/PM1 cell inhibition ratio at 5 µM (%) = 65.8 ± 12.4	Inhibition rate of PM1 cell viability at 10 µM (%) = 12.3 ± 7.2<br>Inhibition rate of PM1 cell viability at 5 µM (%) = None	Not reported	Not reported	[85]
**94**	Naringenin	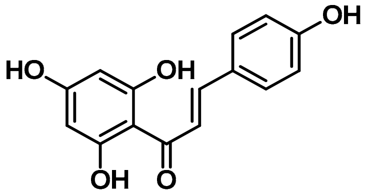	HIV-1	*In vitro*<br>*In silico*	HIV-1 PR: IC_{50} = 33.0 ± 4.59 µg/mL<br>Cat L PR: IC_{50} = >100,000.0 µg/mL<br>HIV-1 PR: E_{dock} = −55.45 kcal/mol<br>Cat L PR: E_{dock} = −45.33 kcal/mol	Not reported	Not reported	HIV-1 protease inhibitor	[48]
**95**	4a (R = 4'-NO_2)	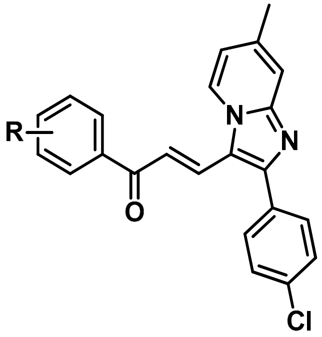	HIV-1<br>HIV-2	*In vitro*<br>*In silico*	HIV-1(III_B) and HIV-2 (ROD)/MT-4 cell: EC_{50} = >96.42 µg/mL /SI: <1<br>PDB ID-1REV: E_{dock} = −11.8 kcal/mol<br>PDB ID-3EBZ: E_{dock} = −9.8 kcal/mol	MT-4 cell: CC_{50} = 96.42 µg/mL	Not reported	HIV-1 reverse transcriptase and HIV-2 protease inhibitor	[82]
**96**	4b (R = 2'-Cl)		HIV-1<br>HIV-2	*In vitro*<br>*In silico*	HIV-1(III_B) and HIV-2 (ROD)/MT-4 cell: EC_{50} = >125.00 µg/mL /SI: <1<br>PDB ID-1REV: E_{dock} = −11.7 kcal/mol<br>PDB ID-3EBZ: E_{dock} = −9.8 kcal/mol	MT-4 cell: CC_{50} = >125.00 µg/mL	Not reported	HIV-1 reverse transcriptase and HIV-2 protease inhibitor	[82]
**97**	4e (R = 4'-Cl)		HIV-1<br>HIV-2	*In vitro*<br>*In silico*	HIV-1(III_B) and HIV-2 (ROD)/MT-4 cell: EC_{50} = >94.37 µg/mL /SI: <1<br>PDB ID-1REV: E_{dock} = −11.0 kcal/mol<br>PDB ID-3EBZ: E_{dock} = −9.7 kcal/mol	MT-4 cell: CC_{50} = >94.37 µg/mL	Not reported	HIV-1 reverse transcriptase and HIV-2 protease inhibitor	[82]
**98**	4c (R = H)		HIV-1<br>HIV-2	*In vitro*<br>*In silico*	HIV-1(III_B) and HIV-2 (ROD)/MT-4 cell: EC_{50} = >125.00 µg/mL /SI: <1<br>PDB ID-1REV: E_{dock} = −9.8 kcal/mol<br>PDB ID-3EBZ: E_{dock} = −8.8 kcal/mol	MT-4 cell: CC_{50} = >125.00 µg/mL	Not reported	HIV-1 reverse transcriptase and HIV-2 protease inhibitor	[82]

S/N	Chalcones	Structure	Virus	Study Model	Inhibitory Concentration IC$_{50}$/EC$_{50}$ Docking energy/score Selectivity/Therapeutic index SI/TI	Cytotoxic Concentration CC$_{50}$	Inhibition Stage	Target / Mode of action	Ref
**99**	4d (R = Me)	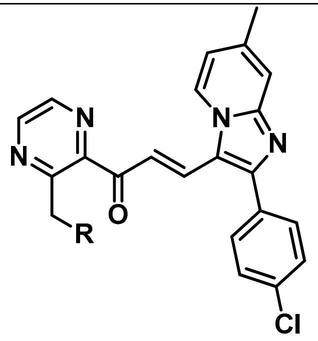	HIV-1 HIV-2	*In vitro* *In silico*	HIV-1(III$_B$) and HIV-2 (ROD)/MT-4 cell: EC$_{50}$ = >125.00 µg/mL /SI: <1 PDB ID-1REV: E_{dock} = −9.8 kcal/mol PDB ID-3EBZ: E_{dock} = −8.9 kcal/mol	MT-4 cell: CC$_{50}$ = >125.00 µg/mL	Not reported	HIV-1 reverse transcriptase and HIV-2 protease inhibitor	[82]
**100**	CHA-12	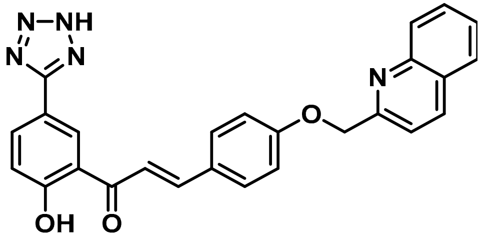	SARS-CoV-2	*In silico*	3CL^{pro} PDB ID-6M2N: ΔGb = −44.03 kcal/mol PL^{pro} PDB ID-7JN2: ΔGb = −35.81 kcal/mol	Not reported	Viral replication	3CL and PL protease inhibitor	[91]
**101**	CHA-37	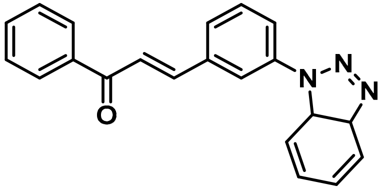	SARS-CoV-2	*In silico*	3CL^{pro} PDB ID-6M2N: ΔGb = −25.05 kcal/mol PL^{pro} PDB ID-7JN2: ΔGb = −21.23 kcal/mol	Not reported	Viral replication	3CL and PL protease inhibitor	[91]
**102**	CHA-378	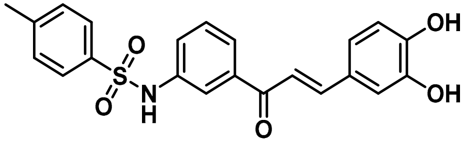	SARS-CoV-2	*In silico*	3CL^{pro} PDB ID-6M2N: ΔGb = −24.98 kcal/mol PL^{pro} PDB ID-7JN2: ΔGb = −20.52 kcal/mol	Not reported	Viral replication	3CL and PL protease inhibitor	[91]
**103**	CHA-384	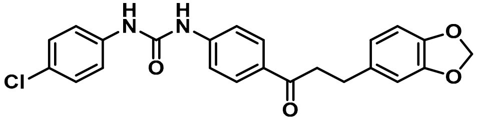	SARS-CoV-2	*In silico*	3CL^{pro} PDB ID-6M2N: ΔGb = −40.05 kcal/mol	Not reported	Viral replication	3CL protease inhibitor	[91]

S/N	Chalcones	Structure	Virus	Study Model	Inhibitory Concentration IC_{50}/EC_{50}<br>Docking energy/score<br>Selectivity/Therapeutic index SI/TI	Cytotoxic Concentration CC_{50}	Inhibition Stage	Target / Mode of action	Ref
**104**	PAACN	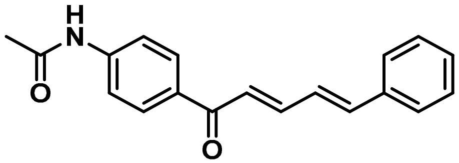	SARS-CoV-2	*In silico*	M^{pro} PDB ID-6LU7: E_{dock} = −6.1 kcal/mol /RMSD = 1.853 Å<br>SPIKE PDB ID-6MOJ: E_{dock} = −6.9 kcal/mol /RMSD = 1.590 Å<br>ACE2 PDB ID-6MOJ: E_{dock} = −7.4 kcal/mol /RMSD = 1.866 Å	Not reported	Entry stage and viral replication	Main protease, SPIKE, and ACE2 inhibitor	[92]
**105**	PAAPA	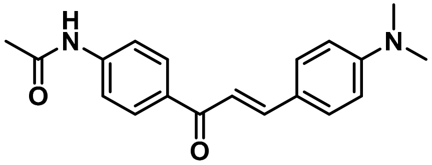	SARS-CoV-2	*In silico*	M^{pro} PDB ID-6LU7: E_{dock} = −5.5 kcal/mol /RMSD = 1.822 Å<br>Methyltransferase PDB ID-6WKQ: E_{dock} = −8.0 kcal/mol /RMSD = 1.930 Å<br>SPIKE PDB ID-6MOJ: E_{dock} = −6.6 kcal/mol /RMSD = 1.168 Å<br>ACE2 PDB ID-6MOJ: E_{dock} = −8.0 kcal/mol /RMSD = 1.355 Å	Not reported	Entry stage and viral replication	Main protease, Methyltransferase, SPIKE, and ACE2 inhibitor	[92]
**106**	PAAPF	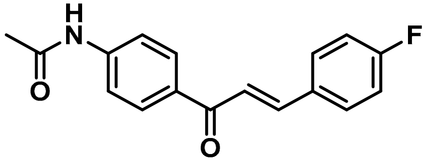	SARS-CoV-2	*In silico*	M^{pro} PDB ID-6LU7: E_{dock} = −5.6 kcal/mol /RMSD = 1.721 Å<br>Methyltransferase PDB ID-6WKQ: E_{dock} = −8.2 kcal/mol /RMSD = 1.947 Å<br>SPIKE PDB ID-6MOJ: E_{dock} = −7.0 kcal/mol /RMSD = 1.759 Å<br>ACE2 PDB ID-6MOJ: E_{dock} = −7.7 kcal/mol /RMSD = 1.658 Å	Not reported	Entry stage and viral replication	Main protease, Methyltransferase, SPIKE, and ACE2 inhibitor	[92]
**107**	A4	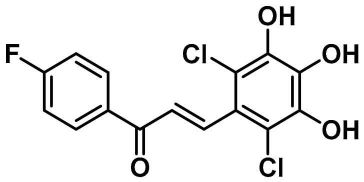	SARS-CoV-2	*In vitro*<br>*In silico*	$3CL^{pro}$: IC_{50} = 83.2 nM<br>SARS-CoV-2 replicon: EC_{50} = 19.9 µM<br>PDB ID: 7U29 ($3CL^{pro}$) /D_{score} = −8.6 kcal/mol<br>PDB ID: 7XAM ($3CL^{pro}$) /D_{score} = −5.4 kcal/mol<br>PDB ID: 7AGA ($3CL^{pro}$) /D_{score} = −7.7 kcal/mol	Huh7 cell: CC_{50} = 41.2 µM	Viral replication	3CL protease noncovalent mixed inhibitor	[93]
**108**	A7	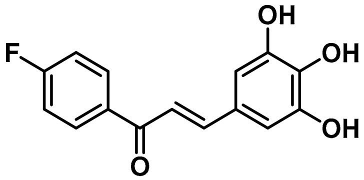	SARS-CoV-2	*In vitro*<br>*In silico*	$3CL^{pro}$: IC_{50} = 261.3 nM<br>SARS-CoV-2 replicon: EC_{50} = 11.7 µM<br>PDB ID: 7U29 ($3CL^{pro}$) /D_{score} = −8.0 kcal/mol<br>PDB ID: 7XAM ($3CL^{pro}$) /D_{score} = −4.8 kcal/mol<br>PDB ID: 7AGA ($3CL^{pro}$) /D_{score} = −4.6 kcal/mol	Huh7 cell: CC_{50} = >50 µM	Viral replication	3CL protease covalent inhibitor	[93]

S/N	Chalcones	Structure	Virus	Study Model	Inhibitory Concentration IC_{50}/EC_{50} Docking energy/score Selectivity/Therapeutic index SI/TI	Cytotoxic Concentration CC_{50}	Inhibition Stage	Target / Mode of action	Ref
**109**	B5	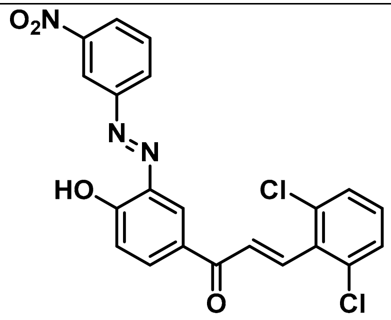	SARS-Cov-2	*In silico*	$3CL^{pro}$: $D_{score} = -6.235$	Not reported	Viral replication	3CL protease inhibitor	[96]
**110**	B6	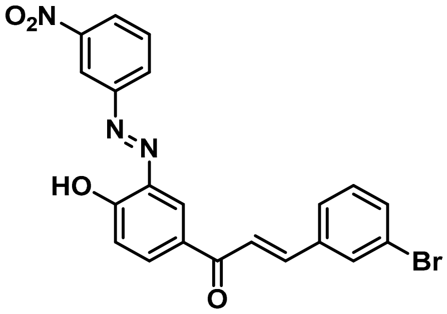	SARS-CoV-2	*In silico*	$3CL^{pro}$: $D_{score} = -5.823$	Not reported	Viral replication	3CL protease inhibitor	[96]
**111**	B8	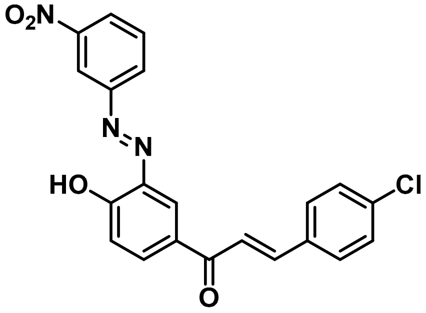	SARS-CoV-2	*In silico*	$3CL^{pro}$: $D_{score} = -5.832$	Not reported	Viral replication	3CL protease inhibitor	[96]
**112**	4-hydroxychalcone	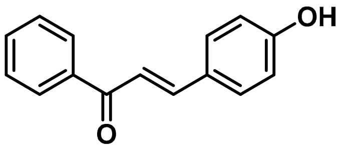	HCoV-OC43	*In vitro* *In vivo* *In silico*	HCoV-OC43/RD: $IC_{50} = 1.83 \pm 0.17$ µM /SI = 13.46 20 mg/kg increases the survival rate by 75% PDB ID: 5D41 (EGFR)/ $E_{dock} = -6.304$ kcal/mol	RD cell: $CC_{50} = 24.63 \pm 2.19$ µM	Early stage of viral replication	EGFR/AKT/ERK1/2 signaling pathway inhibitor	[99]

S/ N	Chalcones	Structure	Virus	Study Model	Inhibitory Concentration IC_{50}/EC_{50} Docking energy/score Selectivity/Therapeutic index SI/TI	Cytotoxic Concentration CC_{50}	Inhibition Stage	Target / Mode of action	Ref
**113**	Kuraridin	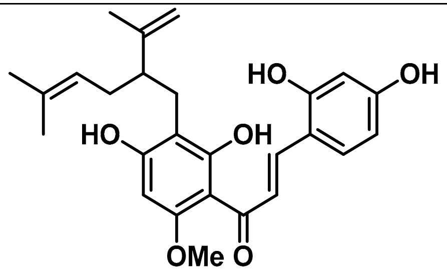	HRV	*In vitro*	HRV1 (T1L) /TF-104 cell: EC_{50} = 62.0 ± 1.8 μM /SI = 4.90<br>HRV2 (T2J) /TF-104 cell: EC_{50} = 29.4 ± 2.7 μM /SI = 10.28<br>HRV3 (T3D) /TF-104 cell: EC_{50} = 14.4 ± 1.2 μM /SI = 20.98<br>PRV (KRP113) /TF-104 cell: EC_{50} = 14.0 ± 4.1 μM /SI = 21.59	TF-104 cell: CC_{50} = 302.2 ± 1.6	Viral entry and late stage of viral replication	Sigma-1 protein inhibitor	[49]

IC_{50} = half-maximal inhibitory concentration; EC_{50} = half-maximal effective concentration; CC_{50} = 50% cytotoxic concentration; SI = selectivity index or TI = therapeutic index (CC_{50}/IC_{50}); E_{dock} = docking energy; D_{score} = docking score; K_i = inhibition constant; K_d = dissociation constant.

Table S2. SMILES strings used for *in silico* ADMET profiling.

1.	OC1=C(C(/C=C/C2=CC=C(O)C=C2)=O)C=CC(O)=C1
2.	O=C(/C=C/C1=CC=C(O)C=C1)C2=C(O)C(C/C=C(C)/C)=C(O)C=C2
3.	O=C(/C=C/C1=CC=C(O)C=C1)C2=C(OC)C=C(O)C(C/C=C(C)/C)=C2O
4.	O=C(/C=C/C1=C(OC)C=C(O)C=C1)C2=CC=C(O)C=C2
5.	O=C(/C=C/C1=C(OC)C=C(O)C(C(C)(C)C=C)=C1)C2=CC=C(O)C=C2
6.	O=C(/C=C/C1=C(OC)C(O)=C(O)C=C1)C2=CC=C(O)C=C2
7.	O=C(/C=C/C1=CC=C(O)C=C1)C2=C(O)C(C/C=C(C)/C)=C(OC)C=C2
8.	O=C(/C=C/C1=CC=C(O)C=C1)C2=C(O)C(C/C=C(C)/CC/C=C(C)/C)=C(O)C=C2
9.	O=C(/C=C/C1=CC=C(O)C=C1)C2=C(O)C(C/C=C(C)/CCC(O)C(C)=C)=C(O)C=C2
10.	O=C(/C=C/C1=CC=C(O)C=C1)C2=C(O)C(CC(O)C(C)=C)=C(OC)C=C2
11.	O=C(/C=C/C1=CC=C(O)C=C1)C2=C(O)C(CC(OO)C(C)=C)=C(OC)C=C2
12.	O=C(/C=C/C1=CC=C(O)C=C1)C2=C(O)C(C/C=C(C)/CC/C=C(C)/C)=C(OC)C=C2
13.	O=C(/C=C/C1=CC=C(O)C=C1)C2=C(O)C(C/C=C(C)/CCC(C(C)=C)O)=C(OC)C=C2
14.	OC1=C(C(/C=C/C2=CC=C(O)C=C2)=O)C=CC(O)=C1C/C=C(C)/CCC(OC)OC
15.	O=C(C1=C(OC)C=C(O)C(CC(C/C=C(C)\C)C(C)=C)=C1O)/C=C/C2=CC=C(O)C=C2O
